# Antitumor Activity of Rapamycin in a Phase I Trial for Patients with Recurrent PTEN-Deficient Glioblastoma

**DOI:** 10.1371/journal.pmed.0050008

**Published:** 2008-01-22

**Authors:** Tim F Cloughesy, Koji Yoshimoto, Phioanh Nghiemphu, Kevin Brown, Julie Dang, Shaojun Zhu, Teli Hsueh, Yinan Chen, Wei Wang, David Youngkin, Linda Liau, Neil Martin, Don Becker, Marvin Bergsneider, Albert Lai, Richard Green, Tom Oglesby, Michael Koleto, Jeff Trent, Steve Horvath, Paul S Mischel, Ingo K Mellinghoff, Charles L Sawyers

**Affiliations:** 1 Department of Neurology, Jonsson Comprehensive Cancer Center, David Geffen School of Medicine, University of California Los Angeles, Los Angeles, California, United States of America; 2 Department of Pathology and Laboratory Medicine, Jonsson Comprehensive Cancer Center, David Geffen School of Medicine, University of California Los Angeles, Los Angeles, California, United States of America; 3 Translational Genomics Research Institute, Phoenix, Arizona, United States of America; 4 Department of Molecular and Medical Pharmacology, Jonsson Comprehensive Cancer Center, David Geffen School of Medicine, University of California Los Angeles, Los Angeles, California, United States of America; 5 Taylor Technology, Princeton, New Jersey, United States of America; 6 Department of Neurosurgery, Jonsson Comprehensive Cancer Center, David Geffen School of Medicine, University of California Los Angeles, Los Angeles, California, United States of America; 7 Department of Neurology, Kaiser Permanente; Los Angeles, California, United States of America; 8 Department of Biostatistics and Human Genetics, Jonsson Comprehensive Cancer Center, David Geffen School of Medicine, University of California Los Angeles, Los Angeles, California, United States of America; 9 Department of Human Oncology and Pathogenesis Program, Memorial Sloan Kettering Cancer Center, New York, New York, United States of America; Brain Tumor Institute, Cleveland Clinic Foundation, United States of America

## Abstract

**Background:**

There is much discussion in the cancer drug development community about how to incorporate molecular tools into early-stage clinical trials to assess target modulation, measure anti-tumor activity, and enrich the clinical trial population for patients who are more likely to benefit. Small, molecularly focused clinical studies offer the promise of the early definition of optimal biologic dose and patient population.

**Methods and Findings:**

Based on preclinical evidence that phosphatase and tensin homolog deleted on Chromosome 10 (PTEN) loss sensitizes tumors to the inhibition of mammalian target of rapamycin (mTOR), we conducted a proof-of-concept Phase I neoadjuvant trial of rapamycin in patients with recurrent glioblastoma, whose tumors lacked expression of the tumor suppressor PTEN. We aimed to assess the safety profile of daily rapamycin in patients with glioma, define the dose of rapamycin required for mTOR inhibition in tumor tissue, and evaluate the antiproliferative activity of rapamycin in PTEN-deficient glioblastoma. Although intratumoral rapamycin concentrations that were sufficient to inhibit mTOR in vitro were achieved in all patients, the magnitude of mTOR inhibition in tumor cells (measured by reduced ribosomal S6 protein phosphorylation) varied substantially. Tumor cell proliferation (measured by Ki-67 staining) was dramatically reduced in seven of 14 patients after 1 wk of rapamycin treatment and was associated with the magnitude of mTOR inhibition (*p* = 0.0047, Fisher exact test) but not the intratumoral rapamycin concentration. Tumor cells harvested from the Ki-67 nonresponders retained sensitivity to rapamycin ex vivo, indicating that clinical resistance to biochemical mTOR inhibition was not cell-intrinsic. Rapamycin treatment led to Akt activation in seven patients, presumably due to loss of negative feedback, and this activation was associated with shorter time-to-progression during post-surgical maintenance rapamycin therapy (*p* < 0.05, Logrank test).

**Conclusions:**

Rapamycin has anticancer activity in PTEN-deficient glioblastoma and warrants further clinical study alone or in combination with PI3K pathway inhibitors. The short-term treatment endpoints used in this neoadjuvant trial design identified the importance of monitoring target inhibition and negative feedback to guide future clinical development.

Trial registration: http://www.ClinicalTrials.gov (#NCT00047073).

## Abstract 2

In a Phase I clinical trial Charles Sawyers and colleagues investigated the role of rapamycin in patients with PTEN-deficient glioblastoma.

## Editors' Summary

**Background.:**

Glioblastoma is a highly malignant tumor of the brain. As with other tumors, it can result from a number of different molecular changes. Traditional chemotherapy does little more than contain these tumors, and cannot cure it. An alternative approach to the treatment of such tumors is to target specific molecular changes in the tumor. Obviously such targeted treatment will work only in patients who have the specific molecular defect being targeted. Hence, traditional clinical trials, which include a large variety of different patients and tumors with different genetic changes, may be an inappropriate way to test how effective targeted treatments are.

One specific change that has been identified in around 40% of patients with glioblastoma is inactivation of a gene known as *PTEN*, which acts as a tumor suppressor gene. When *PTEN* is inactivated it has previously been shown to make cells more sensitive to a class of drugs known as mTOR inhibitors—one of which is rapamycin (trade name Sirolimus). mTOR is a protein that is involved in the regulation of a number of cellular processes including growth and proliferation. Drugs active against mTOR are currently being tested for effectiveness against other cancers and as immunosuppressive agents.

**Why Was This Study Done?:**

This was a Phase I study—that is, the earliest type of a drug study that is done in humans—which aimed to look at the safety of rapamycin in a selected group of patients who were undergoing surgery after recurrence of glioblastoma, and whose tumors did not express PTEN. In addition, the authors also wanted to assess the feasibility of incorporating detailed molecular studies of the action of this drug into such a Phase I study and whether these molecular studies could predict whether patients were more or less likely to respond to rapamycin.

**What Did the Researchers Do and Find?:**

A total of 15 patients were treated with rapamycin at differing doses for one week before surgery and then again after surgery until there was evidence that the tumors were progressing. There was no evidence of very severe toxicity in any of the patients, though there were some adverse effects that required treatment. When samples from the patients were tested after surgery, seven of them showed a reduction in how rapidly the tumor cells divided, and this reduction was associated with how much inhibition there was of mTOR. Two of these patients showed evidence on scans of a reduction in tumor mass. Cells from tumors that appeared resistant to rapamycin in patients were sensitive to rapamycin in tissue culture, suggesting that the lack of response was due to the drug not being able to penetrate the tumor. A second, unfortunate effect of rapamycin was to cause activation of another intracellular protein, Akt, in some patients; when this activation occurred, patients had a shorter time between surgery and a return of their disease.

**What Do These Findings Mean?:**

The detailed molecular studies within this Phase I trial allow a better understanding of how this targeted drug works. These findings suggest that the rapamycin can reduce the proliferation rate of glioblastoma cells, and that this reduction appears to be related to how well the drug is able to penetrate the tumor and inhibit mTOR. However, in some patients the activation of a second pathway can speed up the course of the disease, so further trials should incorporate inhibitors of this second pathway.

**Additional Information.:**

Please access these Web sites via the online version of this summary at http://dx.doi.org/10.1371/journal.pmed.0050008.

- The US National Cancer Institute provides information on all aspects of cancer (in English and Spanish)
- The UK charity Cancerbackup  provides information on brain tumors
- Wikipedia has a page on mTOR (note that Wikipedia is a free online encyclopedia that anyone can edit; available in several languages)

## Introduction

When a new cancer drug first enters the clinic, its development typically proceeds empirically by defining the maximum tolerated dose, then assessing clinical activity across a range of diseases. In the era of molecularly targeted cancer therapy, this approach has been questioned, because it is anticipated that these agents will be effective primarily in those patients whose tumors are dependent on the molecular lesion that is specifically targeted by the new agent [1–3]. However, target-focused clinical development is challenging, because clearly defined, validated molecular criteria to select patients for clinical trials must be established. Inability to access tumor tissue in most patients with solid tumors presents further difficulties. One approach is to conduct small pilot studies in which the targeted agent is administered to patients prior to a scheduled tumor resection to ensure access to tissue during treatment. Such neoadjuvant studies have been successfully implemented with hormonal agents alone or in combination with kinase inhibitors in breast cancer [4,5]. Current technologies permit analyses of gene copy number, mutation status, and mRNA and protein expression from small tissue samples, thereby allowing for the collection of high–molecular content datasets that can guide further clinical development. We have used this approach to study the targeted agent rapamycin in a molecularly defined subset of patients with recurrent glioblastoma.

Inhibitors of the mammalian target of rapamycin (mTOR) have received regulatory approval as immunosuppressive agents for the treatment of allograft rejection and as antitumor agents for kidney cancer [6,7]. Rapamycin and its analogs (CCI-779, RAD001) have shown antitumor activity across a variety of human cancers in clinical trials, but molecular determinants of drug response are currently unknown [8]. Previous work by our group [9] and others [10–15] demonstrated that mutational activation of the phosphatidyl-inositol-3-kinase (PI3K) pathway through loss of PTEN (phosphatase and tensin homolog deleted on Chromosome 10) or activation of the serine/threonine kinase Akt sensitizes tumor cells to the antiproliferative activity of mTOR inhibitors in preclinical models. These findings provided the rationale to explore the antitumor activity of mTOR inhibitors in patients with PTEN-deficient tumors.

Glioblastoma is one model disease to address this question, because PTEN inactivation occurs in ∼40% of patients. Furthermore, salvage surgical resection is often part of the clinical management of patients who relapse after standard up-front therapy (which typically consists of surgical resection followed by adjuvant radiation and chemotherapy). This second resection is an opportunity to collect tumor tissue to assess the molecular effects of treatment administered pre-operatively. Indeed, others have used this salvage surgery to define the dose of O6-benzylguanine required to deplete the DNA-repair protein AGT, which is associated with resistance to temozolamide [16]. Importantly, the antitumor effects of mTOR inhibition in many preclinical models are cytostatic, raising the possibility that traditional radiographic clinical endpoints of tumor shrinkage may not be observed. Glioblastoma may be suitable for assessing cytostatic activity, because these tumors are highly proliferative. Therefore, short-term effects of treatment on growth kinetics could be detectable by immunohistochemical analysis. Finally, clinical benefit can be assessed by measuring time-to-tumor progression after surgery. For these reasons, we conducted a neoadjuvant clinical trial of rapamycin in patients with relapsed, PTEN-negative glioblastoma undergoing salvage resection, with the primary goals of defining a dose required for mTOR target inhibition and assessing potential antiproliferative effects on tumor cells.

## Methods

### Participants

This Phase I trial was registered with http://www.ClinicalTrials.gov (#NCT00047073) (see also http://www.cancer.gov/search/ViewClinicalTrials.aspx?cdrid=257255&version=patient&protocolsearchid=3718462). The clinical trial protocol (#02-03-078–11) was approved by the Institutional Review Board of the University of California Los Angeles. Enrollment was restricted to patients with a histological diagnosis of glioblastoma (GBM), radiographic evidence for disease recurrence after standard GBM therapy (surgery, radiation, temozolamide), evidence for PTEN loss in tumor tissue (see below), and no previous mTOR inhibitor therapy. Other enrollment criteria included age > 18 y, Karnofsky performance score (KPS) ≥ 60, life expectancy ≥ 8 wk, adequate bone marrow function (white blood cell [WBC] > 3,000/μl, absolute neutrophil count [ANC] > 2,000/μl, platelets > 100,000/μl, hemoglobin > 10 gm/dl), adequate liver and renal function (serum glutamic oxaloacetic transaminase [SGOT] and bilirubin < 2.5× upper limits of normal, creatinine < 1.5 mg/dl), plasma cholesterol < 350 mg/dl, and plasma triglycerides < 400 mg/dl. Irradiation and/or chemotherapy were discontinued for ≥ 4 wk before trial entry (≥ 6 wk if prior therapy included a nitrosourea compound). All 15 patients enrolled in the clinical trial gave written informed consent to participate in these evaluations.

### Interventions

Fifteen patients with PTEN-deficient tumors, who also met all other eligibility criteria, were enrolled at the time of tumor recurrence and received neoadjuvant oral daily rapamycin (2 mg, 5 mg, or 10 mg/d) for approximately 1 wk (median: 6 d, mean: 7.5 d) prior to salvage surgical resection (S2). After recovery from surgery, patients resumed daily rapamycin treatment at the neoadjuvant dose until clinical and/or radiographic evidence for tumor progression was found.

### Objectives

The primary goals of this phase I trial were as follows: (1) to define in PTEN-deficient glioblastoma the dose of rapamycin required for mTOR inhibition; (2) to establish in PTEN-deficient glioblastoma the antiproliferative activity of rapamycin; and (3) to define the safety profile of daily rapamycin in patients with glioma.

### Outcomes

#### Effect of rapamycin on mTOR activity and tumor cell proliferation in tumor tissue.

To quantify mTOR activity in matched S1 and S2 samples, we measured phosphorylation of S6 ribosomal protein by immunohistochemistry using two distinct phosphosite antibodies directed against Ser235/236 or Ser240/244. To determine if 7 d of rapamycin treatment had any antitumor activity, we assessed the proliferation rate of matched S1 and S2 samples by measuring the Ki-67 labeling index. Immunohistochemistry (IHC) scoring is described in detail in Text S1.

#### Rapamycin concentrations in peripheral blood and tumor tissue.

Determination of rapamycin concentrations in peripheral whole blood was performed by the UCLA Medical Center Clinical Laboratory using high-performance liquid chromatography/mass spectrometry (HPLC/MS). Quantification of intratumoral rapamycin levels was performed by SFBC Taylor (Princeton, New Jersey) using the following protocol: Fresh frozen tissue samples ranging in mass between 50 and 250 mg were homogenized in water to yield a tissue homogenate concentration of 0.200 g tissue/ml. Rapamycin-free control tissue was homogenized in a similar fashion. Calibrants were prepared from aliquots of the control tissue homogenate pool by spiking with rapamycin to appropriate levels. Desmethoxyrapamycin was used as an internal standard and was spiked into 1.00 ml aliquots of samples and standards (if needed samples were prediluted to 1.00 ml with control tissue homogenate). The homogenates were then extracted with 1-chlorobutane. The extracts were isolated, dried, and reconstituted to a final volume of 100 μl. Forty μl of the extracts were analyzed by liquid chromatography/atmospheric pressure chemical ionization/mass spectrometry/mass spectrometry (LC/APCI/MS/MS) in positive ion mode. Chromatography was performed at a temperature of 50 °C on a YMC ODS-AQ C18 column (Waters), 2.0 × 100 mm, 5 μm column using a Paradigm pump (Michrom Bioresources) Mobile phases were 20 mM ammonium acetate and 0.0005% acetic acid in water, 20 mM ammonium acetate and 0.0005% acetic acid in methanol. Detection was performed on a Finnigan TSQ Quantum Ultra AM mass spectrometer.

#### Tolerability of neoadjuvant and postoperative rapamycin.

Adverse events were evaluated according to the National Cancer Institute Common Toxicity Criteria, version 2 (http://ctep.info.nih.gov/reporting/index.html).

#### Genomic studies.

Tumor cell DNA was isolated from microdissected fresh frozen clinical tumor samples using the Qiagen DNeasy Kit (Qiagen). Bidirectional full length sequencing of *PTEN* (exons 2–9) was performed by Agencourt, and sequence traces were analysed using Mutation Surveyor software (Softgenetics). For gene copy number determination, labeled tumor DNA was hybridized to Agilent 44A comparative genomic hybridization (CGH) microarrays consisting of ∼40,000 oligonucleotide probes (Agilent Technologies) and scanned on an Agilent DNA microarray scanner. Raw log_2_ ratio data were calculated using Agilent Feature Extraction 9.1 software. Log_2_ ratios for PTEN were generating using the ADM1 aberration calling algorithm implemented in Agilent's CGH Analytics 3.4 software. A detailed description of array-CGH methodology is provided in Text S1.

### Sample Size

One hundred and sixty five patients were screened for PTEN status after initial surgical resection, then followed until relapse. Fifteen patients whose initial surgical samples stained negative for PTEN by immunohistochemistry were treated with rapamycin for about 1 wk prior to a planned salvage surgical resection. Tumor samples from nine glioblastoma patients who underwent S1 and S2 surgeries at UCLA but did not receive rapamycin served as controls for changes in phosphoS6 and Ki-67 staining ratio from S1 to S2. For additional comparisons, Ki-67 staining was also measured in S2 samples from an additional 12 patients whose tumors showed reduced PTEN staining but who did not receive rapamycin. (Matched S1 samples were not available for this latter group of tumors).

### Statistical Methods

Since time to progression (TTP) was uncensored, we were able to use (multivariate) linear regression models to relate TTP to the molecular and clinical variables. Since TTP was highly skewed, we log transformed it to satisfy the normality assumption of a linear regression model. We used Kaplan Meier plots to visualize the TTP distributions for different patient strata and used the log rank to test for differences. To test for median differences across different patient groups, we used nonparametric group comparison tests (Wilcoxon, Kruskal Wallis test). For example, we used the Wilcoxon test to compare Ki67% among the different patient and control groups. We used the Fisher exact test to test the independence of rows and columns in a contingency table.

### Reagents

The following antibodies were used for IHC: anti-PTEN (6H2.1, #ABM-2052, Cascade BioScience; 1:400 dilution), anti-Ki-67 (MIB-1, M7240, DakoCytomation; 1:100 Dilution), anti-phospho Ser235/236 S6 (91B2, #4857, Cell Signaling; 1:50 dilution), anti phospho Ser240/244 S6 (#2215, Cell Signaling; 1:200 dilution), anti-phospho-PRAS 40 (#44–1100G, Biosource; 1:200 dilution), anti-phospho Ser473 Akt (736E11, #3787, Cell Signaling; 1:50 dilution), anti-phospho Thr389 p70 S6 Kinase (#9205, Cell Signaling; 1:100 Dilution), and anti phospho Ser1108 eIF4G at 1:400 (#2441, Cell Signaling; 1:400 dilution). Antigen retrieval was performed using 0.01 M citrate buffer, pH 6.0 for 30 min in an oven and peroxidase activity was quenched with 3% hydrogen peroxide in water. Primary antibodies were diluted in PBS /2% bovine serum albumin/2% normal horse serum (for anti-PTEN) or TBS/0.1% Tween/5% normal goat serum (for phosphosite-specific antibodies) and applied overnight at 4 °C. Biotinylated secondary antibodies (Vector) were applied at 1:200 dilution for 45 min, and the avidin-biotin complex (Elite ABC, Vector) for 30 min. Vector NovaRed was used as the enzyme substrate to visualize specific antibody localization. For Ki-67 staining, antigen retrieval was performed with a 1mM EDTA buffer (pH 8.0) for 13 min in a pressure cooker microwave (power level: 80%). All slides were counterstained with Harris hematoxylin.

## Results

### Design of a Neoadjuvant Glioblastoma Trial with Short-Term, Tissue-Based Endpoints

**Figure 1:**
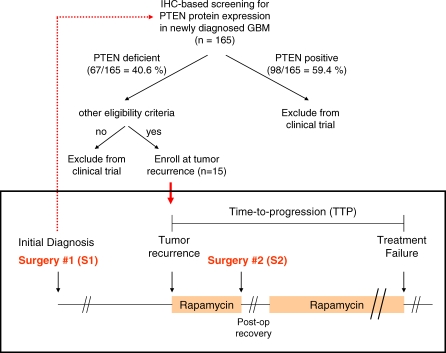
Clinical Trial Design Enrollment in the Phase I clinical trial was restricted to patients whose initial tumor resection (“surgery 1”) specimen was PTEN-deficient by immunohistochemistry. Patients were enrolled after failing standard therapy with radiation and chemotherapy (i.e., “tumor recurrence”). Prior to the scheduled salvage tumor resection (“surgery 2”), patients received a short course (mean: 7.5 d) of oral rapamycin. Rapamycin was resumed after recovery from surgery until patients developed clinical and/or radiographic evidence of treatment failure. The effects of rapamycin on tumor cell proliferation and mTOR signaling in tumor tissue were determined by comparing the tumor tissue collected during salvage resection (“surgery 2”) with a sample of the same tumor collected during the initial tumor resection (“surgery 1”). Time-to-progression (TTP) was defined as the interval between start of rapamycin therapy and postoperative treatment failure.

Our primary motive in conducting this single-arm study was to follow up on the compelling preclinical activity of mTOR inhibitors in PTEN-null cancer models by designing a small clinical trial focused on measuring antitumor activity using short-term endpoints. To enhance the probability of success based on the preclinical hypothesis, we restricted enrollment to those patients with recurrent glioblastoma whose tumors had evidence of PTEN loss based on an analysis of tissue obtained from the initial resection (S1) (Figure 1). Eligibility was also limited to those patients scheduled to undergo salvage surgical resection (S2) so that tumor tissue would be available for assessing the endpoints of mTOR inhibition and tumor cell proliferation, as well as intratumoral rapamycin concentrations. By mandating access to pre- and posttreatment samples for each patient, this trial design allows intrapatient comparison of molecular endpoints, thereby enhancing the statistical power to detect changes in a small sample size. To provide confidence that any S1-to-S2 changes could be attributed to rapamycin treatment, we conducted an identical set of measurements using S1 and S2 samples from nine glioblastoma patients who did not receive rapamycin (controls).

Patients whose tumors had PTEN loss were identified using a previously reported semi-quantitative scoring system that evaluates PTEN expression in tumor cells relative to adjacent vascular endothelial cells [17,18]. We screened tumor samples obtained at the time of initial surgery (S1) from 165 glioblastoma patients followed at our institution for subsequent neuro-oncology care. Either complete (43/165) or partial (24/165) loss of PTEN immunoreactivity was shown in 67/165 (40.6 %) of tumors. Fifteen patients with PTEN-deficient tumors, who also met all other eligibility criteria (see Methods, Texts S2 and S3), were enrolled at the time of tumor recurrence and received neoadjuvant oral daily rapamycin (2 mg, 5 mg, or 10 mg per day) for approximately 1 wk (median: 6 d, mean: 7.5 d) (Table 1) prior to salvage surgical resection (S2). Matching S1 and S2 samples were used to evaluate the effects of rapamycin on tumor cell proliferation and mTOR activity. After recovery from surgery, patients resumed daily rapamycin treatment at the neoadjuvant dose until clinical and/or radiographic evidence for tumor progression was found.

**Table 1:**
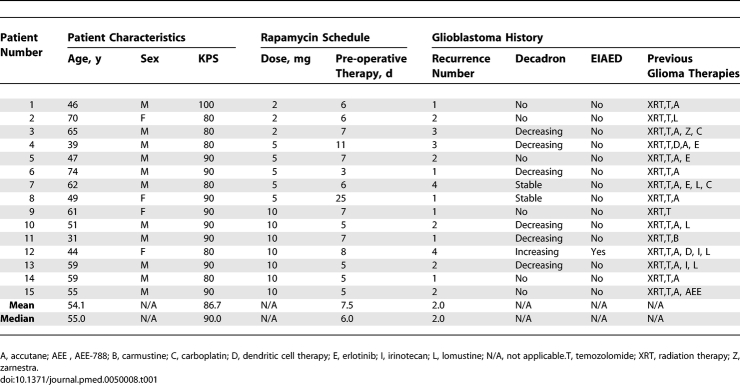
Clinical Characteristics of Rapamycin Study Patients

### Rapamycin Tissue and Blood Levels and S6 Kinase Inhibition in Tumor Tissue

**Figure 2:**
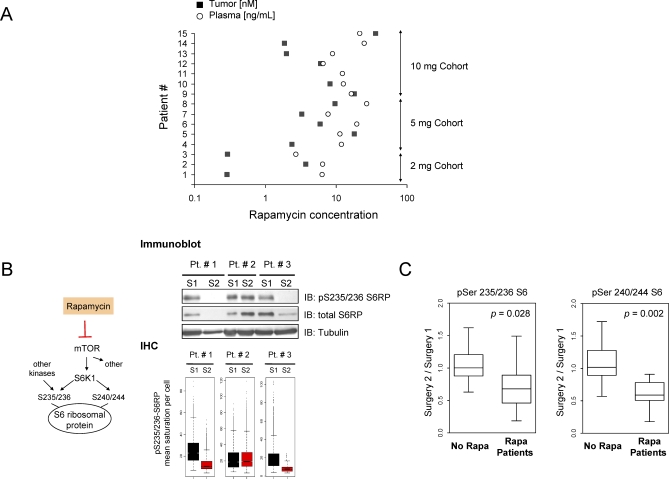
Rapamycin Crosses the Blood–Brain Barrier and Blocks mTOR in Tumor Tissue (A) Rapamycin concentrations in tumor tissue (filled squares) and peripheral blood (empty circles) grouped by rapamycin dose cohorts (2 mg, 5 mg, or 10 mg per os daily). Intratumoral rapamycin concentration for patient 11 could not be determined due to insufficient frozen tumor material. The last preoperative dose of rapamycin was given on the day of craniotomy and peripheral blood was collected within 24 h of surgery. (B) Quantification of mTOR activity in tumor tissue by immunohistochemistry. The cartoon to the left depicts the S6 kinase 1 branch of the mTOR signaling pathway resulting in phosphorylation of S6 ribosomal protein at serine 235/236 and serine 240/244. The panel to the right shows a comparison between immunoblotting (top) and IHC (bottom) for the determination of S6 phosphorylation in tumor tissue from rapamycin patients 1, 2, and 3. The fold change in serine 235/236 phosphorylation between S2 and S1 for patients 1, 2, and 3 were 0.45, 1.01, and 0.45, respectively (see Figure S2A). (C) Changes in S6 phosphorylation between S2 and S1 (*y*-axis: ratio of S6 phosphorylation in S2 sample to S6 phosphorylation in S1 sample) for all patients for whom matched S1 and S2 samples were available (14/15 rapamycin patients and 9/9 patients who did not receive rapamycin). S6 phosphorylation was determined by IHC using phosphosite-specific antibodies against serine 235/236 (left) and serine 240/244 (right). Please see Figures S1 and S2 for details regarding IHC scoring method and results for individual tumors. *p*-values for the difference in mean S2/S1 ratios for each group were determined using the Kruskal Wallace test.

Because rapamycin is a macrolide natural product whose size could prevent distribution across the blood–brain barrier, we measured rapamycin concentrations by mass spectrometry in an aliquot of tumor tissue obtained at S2. Rapamycin was detected in 14 of 14 tumors (insufficient tissue was available from patient 11) at concentrations ranging from 0.3–36.3 nM (Figure 2A). Rapamycin concentrations known to confer antiproliferative activity in PTEN-null cell lines in vitro are typically ∼1 nM [9,11].

To quantify mTOR activity, we measured phosphorylation of S6 ribosomal protein by immunohistochemistry. S6 is a direct substrate of S6 kinase 1, a downstream effector of mTOR action, and has been widely used as a pharmacodynamic readout of mTOR inhibition in preclinical studies. To ensure specificity, we used two distinct phosphosite antibodies directed against Ser235/236 or Ser240/244 (Figure 2B). Both sites are directly phosphorylated by S6 kinase 1, but Ser235/236 can also be phosphorylated by p90 ribosomal S6 kinase (RSK) and other kinases [19]. All measurements were quantified by digital readout of 2,500–5,000 tumor cells per slide cut from paraffin-embedded tissue (Figure S1). We attempted to assess mTOR activity using phosphosite-specific antibodies against Thr389 of S6 kinase 1, a direct mTOR site, and eukaryotic initiation factor 4G (serine 1108), but the performance characteristics of these antibodies on paraffin sections, in our hands, were inadequate for reliable quantification (unpublished data).

For the three patients in the 2-mg cohort, sufficient frozen tissue from S1 and S2 was available to directly compare immunoblot and IHC measures of S6 phosphorylation (Figure 2B). Although the magnitude of pS6 reduction measured by immunoblot was more dramatic, both methods were in agreement and thereby provide reassurance that the IHC approach could be used across the sample set. Total ribosomal S6 levels were also reduced in patients 1 and 3, consistent with the fact that translation of S6 is mTOR-dependent.

When examined in aggregate, the level of S6 phosphorylation in S2 samples from all three cohorts was reduced at both phosphosites compared to matched S1 samples (S2/S1 ratio < 1.0, Figure 2C). In contrast, a similar analysis of S1 and S2 samples collected from the nine glioblastoma patients who did not receive rapamycin showed no change in S6 phosphorylation. In comparing the performance of the two antibodies, it is noteworthy that three patients had an increase (or no change) in S6 phosphorylation in the S2 sample when measured using the serine 235/236 antibody (patients 2, 5, and 13) (Figure S2B), whereas the same samples showed reduced S6 phosphorylation using the serine 240/244 antibody (Figure S3A and Table 2). This paradox might be explained by mTOR-independent phosphorylation of the 235/236 site [20,21] and suggests that the 240/244 site may be a preferred endpoint for assessment of mTOR activity. It is also noteworthy that the magnitude of pS6 phosphorylation did not correlate with the intratumoral rapamycin concentration. Indeed, some patients with adequate drug concentrations in tumor tissue had relatively modest reduction reductions in pS6 (e.g., patients 2 , 12, and 15) (Table 2), indicating that some patients/tumors exhibit apparent biochemical resistance to mTOR inhibition (addressed further below).

**Table 2:**
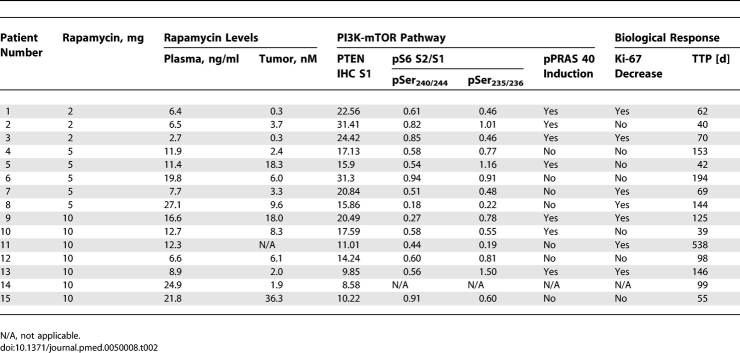
Molecular and Clinical Parameters in the Cohort of Rapamycin-Treated Patients

### Inhibition of Tumor Cell Proliferation Is Correlated with Magnitude of mTOR Inhibition

**Figure 3:**
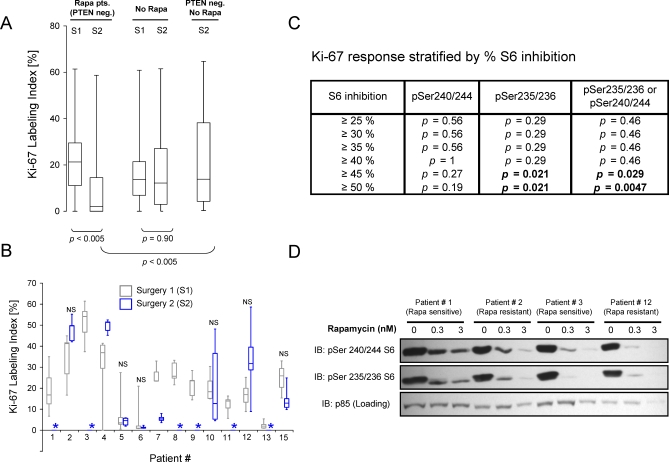
Rapamycin Inhibits Tumor Cell Proliferation in Subsets of PTEN-Deficient GBMs (A) Ki-67 labeling index of GBMs before (S1) and during (S2) rapamycin therapy compared with tumor samples from patients who did not receive rapamycin (see text for detail). The horizontal line inside a box plot shows the median value. The lower and upper end of the box corresponds to the 25th and 75th percentile, respectively. The whiskers extend to the 95% range. The Wilcoxon nonparametric group comparison test was used to calculate *p*-values for the differences between different patient groups. (B) Changes in tumor cell proliferation between S1 and S2 for 14/15 rapamycin patients. The horizontal line inside the box indicates the median value. The lower and upper border of the box corresponds to the 25th and 75th percentile, respectively. The whiskers extend to the 95% range. Paraffin blocks from patient 14 were not sufficient for quantification. The median Ki-67 labeling index of the S2 specimen from patients 1, 3, 8, 9, 11, and 13 was 0 (indicated by asterix). N.S. indicates that the difference in Ki-67 labeling index not statistically significant at the 0.05 level according to the Wilcoxon test. (C) Relationship between the magnitude of S6 inhibition and Ki-67 response in S2 tumor samples from rapamycin-treated patients. *p*-values were determined by Fisher Exact test for different thresholds of pS6 inhibition. (D) Ex vivo rapamycin response of short-term cultures derived from tumors with in vivo S6 response (patients 1 and 3) or resistance (patients 2 and 12). Shown are S6 and p85 (loading) immunoblots of whole cell lysates 8 h after treatment with vehicle, 0.3 nM rapamycin, and 3 nM rapamycin.

To determine if 7 d of rapamycin treatment had any antitumor activity, we assessed the proliferation rate of matched S1 and S2 samples by measuring the Ki-67 labeling index. S2 samples from the rapamycin-treated patients had a significantly lower labeling index than the matched S1 tumors (median: 2.1 % versus 21.3 %, *p* < 0.005 by Wilcoxon test), whereas the same S1/S2 comparison in patients who did not receive rapamycin showed no change (Figure 3A). Because these untreated patients were not selected for PTEN deficiency, we measured proliferation in 12 additional nonstudy patients following salvage resection whose tumors were matched for PTEN status. (Matching S1 samples from these 12 patients were not available for intrapatient comparison.) The Ki-67 labeling index in these PTEN-null S2 samples (no rapamycin) was comparable to that of the S1 and S2 samples from the control patients and significantly higher than the S2 samples from the rapamycin-treated patients (Figure 3A). Remarkably, the reduction in Ki-67 labeling index was attributable to nearly complete inhibition of tumor cell proliferation in half (7/14) of the patients (Figure 3B), suggestive of at least two subgroups (rapamycin-sensitive and rapamycin-resistant) within this patient population.

In examining the molecular determinants of rapamycin sensitivity, we noted that the magnitude of mTOR inhibition was highly correlated with Ki-67 response using a cutoff of >50% inhibition of S6 phosphorylation for at least one of the two examined phosphosites (*p* < 0.0047) (Fisher exact test) (Figure 3C). In comparing the two antibodies, changes in pSer235/236 were statistically more significantly linked to Ki-67 response, even though this site is believed to be less specific for S6K1 activity than pSer240/244 due to phosphorylation by other kinases [20]. This finding may reflect true biological differences in the input to these distinct phosphorylation sites or may be due to the relative sensitivity of the antibodies for detecting quantitative differences in mTOR inhibition. [In our hands, the staining intensity with the pSer240/244 antibody is generally less intense than with the pSer235/236 antibody.]

Whereas this analysis highlights the importance of achieving sufficient mTOR inhibition, it fails to address the fact that adequate intratumoral rapamycin concentrations did not translate into mTOR inhibition in some patients. Such biochemical resistance could be cell-intrinsic (mutation of the drug target, expression of a drug efflux pump in tumor cells, etc.) or host-related (drug bound to serum proteins, sequestration in specific cell types or tissues, etc.). To distinguish between these two categories, we examined the sensitivity of tumor cells removed at S2 from four study patients, two of whom were sensitive and the other two resistant to rapamycin, after short-term propagation in culture. If clinical rapamycin resistance is cell-intrinsic, these cells should be similarly resistant ex vivo, whereas sensitivity should be restored if host mechanisms are at play. Remarkably, S6 phosphorylation was inhibited equally in rapamycin-sensitive (patients 1 and 3) and rapamycin-resistant (patients 2 and 12) samples at 0.3 and 3.0 nM concentrations (Figure 3D), indicating that the failure to inhibit mTOR in these patients is not cell-intrinsic. Rather, the data indicate that delivery of rapamycin to tumor cells is impaired in some patients despite achieving adequate concentrations in resected brain tumor tissue. One possibility, based on the fact that rapamycin is sequestered in red blood cells [22], is that the high intratumoral concentrations of rapamycin observed in these resistant patients reflect red cell pooling in highly vascular tumors. Indeed, tumors from resistant patients showed abundant immunohistochemical staining for the vascular marker CD31 (Figure S4) but the sample size is too small to make definitive conclusions. Alternative explanations include variations in penetration of the blood–brain barrier or tumor hydrostatic pressure among patients.

### Safety Profile of Daily Rapamycin in Patients with Glioma

No grade 3 or 4 toxicities were observed during preoperative rapamycin treatment. Of particular importance, there were no perioperative bleeding complications. Five of 15 patients had grade 3 adverse events (hypokalemia, hypercholesterolemia, and cytopenias) during postoperative rapamycin treatment, which were managed with supportive care and did not require treatment discontinuation (Table S1).

### Impact of Rapamycin-Induced Akt Activation on Clinical Outcome

**Figure 4:**
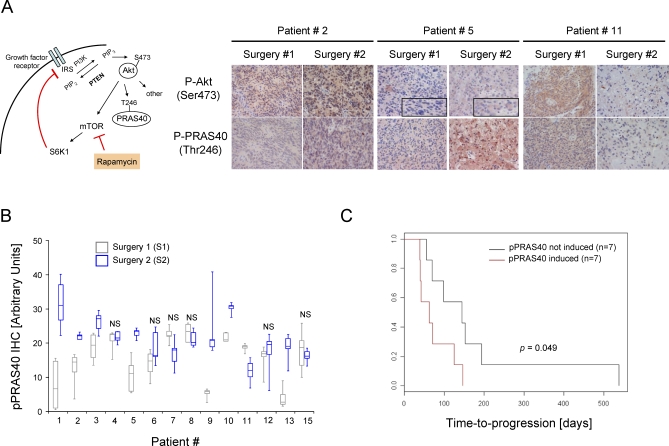
Induction of Akt Signaling in a Subset of Rapamycin-Treated Tumors (A) Determination of Akt activation in tumor tissue during mTOR inhibitor therapy. The left panel is a cartoon illustrating the mTOR/S6K1 dependent feedback loop of the PI3k-Akt pathway. The right panel shows changes in phosphorylation of the Akt-substrate PRAS40 (threonine 246) during rapamycin therapy correspond to changes in Akt phosphorylation (serine 473). Tumors from patients 2 and 5 show an increase in pAkt and pPRAS40 immunostaining during rapamycin treatment, whereas the tumor from patient 11 shows a decrease in the same markers on rapamycin. (B) Changes in PRAS40 phosphorylation between S1 and S2 for 14/15 rapamycin patients. The horizontal line inside the box indicates the median value. The lower and upper border of the box corresponds to the 25th and 75th percentile, respectively. The whiskers extend to the 95% range. Paraffin blocks from patient 14 were not sufficient for quantification. N.S. indicates that the difference in pPRAS40 staining intensity not statistically significant at the 0.05 level according to the Wilcoxon test. (C) Kaplan Meier analysis illustrating the relationship between pPRAS40 induction and time-to-tumor progression.

Physiologic activation of the Akt pathway is regulated, in part, by a negative feedback loop involving phosphorylation of insulin receptor substrate 1 (IRS1) by the mTOR effector molecule S6 kinase 1 (Figure 4A) [23–26]. mTOR inhibition by rapamycin can cancel this negative feedback and activate Akt in some cancer cell lines and tumor samples, but the potential clinical impact is unknown [8,27,28]. We assessed Akt activity in S1 and S2 samples in the rapamycin-treated patients using phosphosite-specific antibodies against the serine/threonine kinase Akt (serine 473) and its downstream substrate PRAS 40 (threonine 246), which serves as a biomarker for Akt activity (Figure 4A). PRAS40 has also been recently shown to inhibit mTOR, and this inhibition is relieved by Akt phosphorylation [29–31]. Seven of 14 (50%) patients had a statistically significant (*p* < 0.05, Wilcoxon test) increase in PRAS40 phosphorylation in their S2 sample (Figure 4B). Of note, one patient (11) had a significant decrease in PRAS40 phosphorylation (and pS473 Akt) at S2 (Figure 4B), which could reflect the potential inhibition of the TORC2 mTOR complex (implicated as the pS473 Akt kinase) by rapamycin after prolonged exposure [32,33].

Because activation of Akt may attenuate the antitumor activity of rapamycin, we examined the relationship between pPRAS 40-induction and time-to-progression during the postsurgical maintenance phase of rapamycin treatment. Induction of pPRAS40 was statistically significantly associated with a shorter time-to-progression (*p* = 0.049, Logrank test) (Figure 4C). Given that only 14 patients led to this *p*-value, it is plausible that a study with more patients could lead to a more statistically robust finding. In contrast, there was no statistically significant relationship between time-to-progression and age (*p* = 0.57), Karnofsky performance status (*p* = 0.91), number of tumor recurrences prior to enrollment (*p* = 0.12), rapamycin levels in tumor (*p* = 0.45) or plasma (*p* = 0.25), inhibition of S6 phosphorylation at serine 240/44 (*p* = 0.42) or serine 235/236 (*p* = 0.65), basal phosphorylation of S6 at serine 240/244 (*p* = 0.24) or serine 235/236 (*p* = 0.54), or Ki-67 response (*p* = 0.42) (all *p*-values determined by Logrank test). pPRAS40 induction remained an independent significant predictor (*p* < 0.05) even after adjusting for age and KPS in a multivariate regression model.

To explore which genetic lesions might be associated with PRAS40 induction during mTOR inhibitor therapy, we hybridized tumor cell DNA collected during rapamycin therapy (S2) to CGH microarrays consisting of ∼40,000 oligonucleotide probes. Overall, our data set showed many chromosomal aberrations typically found in glioblastoma [34], including frequent gains of chromosome 7, loss of chromosome 10, and focal amplifications of EGFR, PDGFRA, CDK4, and MDM2 (Figure S5). Compared to tumors without pPRAS40 induction, tumors with pPRAS40 induction more commonly showed focal amplifications of MDM2 (4/7 versus 1/5) and the receptor tyrosine kinases EGFR or PDGFRA (4/7 versus 1/5) (Figure S6). While these observations lacked statistical power due to the small sample size, it is interesting to note that EGFR has been reported to induce expression of IRS1, the mediator of the negative mTOR/S6K1 feedback loop, in breast cancer cells [35]. Furthermore, PDGFR is an additional target of mTOR negative feedback which can be overcome by enhanced PDGFRβ expression [36].

### Comparison of S1 and S2 PTEN Status

One critical issue that can complicate the use of molecular biomarkers for clinical trial eligibility is whether a tissue sample obtained much earlier than the treatment intervention can be used to make an accurate assessment of the relapsed tumor. We addressed this question by comparing the PTEN status using the S1 and S2 samples from the 15 patients on this study. Although eligibility was determined on the basis of a semiquantitive manual PTEN score [17,18], we later refined our PTEN scoring methodology by using digital image quantification software. In comparing the two scoring methods on 44 archival glioblastoma samples, we found a high Spearman correlation between the two scoring methods (rho = 0.82, *p* = 8.6 × 10^−12^) (Figure S7A). When applied to the study patients, the digital method confirmed that PTEN immunoreactivity in the S1 sample was comparable to that of the S2 sample (all samples scored less than 35) and could be used to infer PTEN status at relapse (Figure S7B).

To explore the molecular basis for loss of PTEN expression, we searched for *PTEN* mutations and/or gene loss in tumor cell DNA microdissected from all available (13/15) frozen tumor samples. 3/13 (23.1%) tumors harbored missense mutations in the *PTEN* coding sequence. One of these mutations (R173C) has previously been described in glioblastoma, and the other two missense mutations (H61Y, V217F) map to codons previously reported in other human cancer samples (http://www.sanger.ac.uk/genetics/CGP/cosmic). 9/13 (69.2 %) tumors showed evidence of DNA copy-number loss (log_2_ ratio ≤ −0.3) at the *PTEN* locus in at least one tumor aliquot, as measured by oligonucleotide array-based comparative genome hybridization (Table S2). At least one *PTEN* allele was thus found to be inactivated in 10/13 (76.9 %) tumors, confirming that our study population was indeed enriched for PTEN deficient glioblastomas. Our failure to identify *PTEN* mutations and/or *PTEN* DNA copy-number loss in some tumors with PTEN deficiency by IHC might be due to epigenetic mechanisms of PTEN silencing, incomplete sequencing coverage of the *PTEN* gene (exon 1 and the 5′ untranslated region [UTR] not sequenced) or the insensitivity of these methods when using heterogeneous tumor samples (the definition of PTEN deficiency used for the IHC test was >20% of tumor cells) [37].

## Discussion

Rapamycin and other mTOR inhibitors have shown great promise as anticancer drugs in a spectrum of preclinical models, but it has been difficult to demonstrate convincing clinical activity in single-agent trials using conventional radiographic and clinical criteria for response [38]. Potential explanations include the largely cytostatic action of these drugs in the laboratory, uncertainty over dose and schedule, and lack of studies to evaluate the drug in subsets of patients most likely to respond based on molecular phenotypes defined preclinically. The goal of this study was to evaluate directly rapamycin in patients whose tumors have defects in PTEN, based on preclinical findings originally generated by our group and others showing mTOR dependence in such models [9–15]. In designing the clinical experiment, we sought to validate the use of a PTEN assay for patient selection, document mTOR inhibition in tumor tissue (of particular importance for brain cancers), and gain preliminary evidence of antitumor activity. Glioblastoma was selected based on the high frequency of PTEN loss (∼40%), the clinical opportunity to collect tumor tissue at the time of salvage surgical resection, and the high proliferative index of these tumors, providing a robust endpoint for assessing antitumor effect. The intent was to generate information that could be used for more focused hypothesis testing in subsequent trials.

In the present study 165 patients were screened for PTEN status after initial surgical resection, then followed until relapse. Fifteen patients whose initial surgical samples stained negative for PTEN by immunohistochemistry were treated with rapamycin for about 1 wk before a planned salvage surgical resection. Short-term effects of rapamycin on mTOR inhibition in tumor cells and on the tumor proliferation index were determined by comparing immunohistochemical measures of these indices in the initial surgical sample (surgery 1 or S1) to the salvage resection sample (surgery 2 or S2). Rapamycin treatment led to substantial inhibition of tumor cell proliferation in seven of 14 patients, which correlated with the greatest magnitude of mTOR inhibition in tumor tissue. As predicted from preclinical studies [27,28], rapamycin also led to the activation of Akt in some cases, and this activation was significantly correlated with shorter time-to-tumor progression.

The primary findings from this neoadjuvant rapamycin trial are evidence of antitumor activity using a short-term endpoint, novel insights into the importance of achieving sufficient target inhibition, and clinical evidence for evaluating combination PI3-kinase/mTOR therapy to address negative feedback. All three findings should guide future clinical development of mTOR inhibitors in this disease. The Ki-67 response data demonstrate that rapamycin has clear antitumor activity in a subset of patients with PTEN loss. In addition to effects on tumor cell proliferation, two patients also had radiographic evidence of response. Patient 8 received an extended course of neoadjuvant rapamycin (25 d) due to an intercurrent upper respiratory infection and had >50% tumor regression by magnetic resonance imaging prior to surgery (Figure S8A). Patient 11 showed continued radiographic improvement during the postoperative phase of rapamycin treatment and died without evidence of tumor recurrence 538 d after starting rapamycin (Figure S8B). The experience with patient 8 might justify a longer neoadjuvant treatment period to gain radiographic response data on all patients in subsequent trials. While our trial was underway, a single-arm phase II study of the mTOR inhibitor CCI-779 reported that 20 of 65 patients with recurrent glioblastoma (36%) had radiographic improvement [39]. Of note, these patients were not evaluated prospectively for PTEN status (no molecular selection criteria), and CCI-779 was delivered weekly rather than daily based on a phase I experience that defined a maximum tolerated dose using this schedule [40,41]. In light of our findings about the magnitude of mTOR inhibition required for response (discussed below), this schedule raises concerns about the presumed lack of target coverage during nontreatment days. Nonetheless, the fact that both trials showed evidence of antitumor activity provides confidence that further investigation of mTOR inhibitors is warranted. The role of PTEN loss in defining sensitivity could be determined using a trial design in which all patients are initially eligible but sufficient numbers of PTEN negative versus PTEN positive are accrued to allow subset analysis.

Although intuitive, the correlation we found between the magnitude of mTOR inhibition and Ki-67 response was not anticipated from preclinical studies. Nearly complete inhibition of S6 phosphorylation is typically achieved with rapamycin treatment in xenografts and other mouse model systems; therefore, most studies of response have focused on defining genetic lesions (Pten, Akt, Tsc, Vhl, etc.) that affect mTOR dependence of tumor cells [38,42]. The surprising finding in this trial is that despite using doses of rapamycin sufficient to give low nM intratumoral levels, such doses do not translate into mTOR inhibition in all patients. Through ex vivo analysis of tumor cells isolated at salvage surgery, we established that resistance in these patients is not cell intrinsic. Consistent with an extrinsic mechanism of rapamycin resistance, our genomic survey of S2 tumor samples failed to identify significant copy-number alterations within genes in the mTOR pathway (FKBP12, S6 kinase 1, RAPTOR, RHEB, Akt) that might explain the observed rapamycin resistance in vivo. This result contrasts with mechanisms of resistance to other kinase inhibitors (in chronic myeloid leukemia, gastrointestinal stromal tumors, and EGFR-dependent lung cancer), which often occurs through point mutations in the kinase target in tumor cells [43] and raises the possibility that a larger fraction of PTEN null glioblastomas could be rapamycin-sensitive if more significant mTOR inhibition could be achieved.

The more challenging question is whether strategies can be developed to improve delivery of rapamycin directly to tumor cells and maximize mTOR inhibition broadly across all patients. Oral delivery of significantly higher daily doses is an unlikely solution due to problems with tolerability (mucositis, thrombocytopenia) seen in other diseases. Invasive approaches such as convection-enhanced delivery or implantation of drug-impregnated wafers have been used to treat glioblastoma patients with chemotherapeutic agents and may be considered. Alternatively, a better understanding of the reason underlying the failure to achieve mTOR inhibition in selected patients could point to a solution. For example, if rapamycin in these patients is sequestered in red cells due to enhanced tumor vascularity, antiangiogenic agents such as bevacizumab (already known to have activity in glioblastoma) [44] may prevent sequestration and allow more efficient drug delivery. Evaluation of all of these approaches requires quantitative assessment of mTOR activity and highlights the need to develop broadly useful clinical tools for quantitative analysis of target inhibition. In the short term, it may be possible to identify the early Ki-67 responders using PET tracers such as 3′-deoxy-3′-18F-fluorothymidine (FLT) that can read out proliferation noninvasively [45]. Although such identification would not itself improve rapamycin delivery to the tumor cells, it could at least identify the subset of tumors in which rapamycin delivery appears to be problematic. Success here would also obviate the need for salvage surgery and could greatly expand eligibility of patients for larger trials.

There seems little doubt from the time-to-progression curves reported here and in the CCI-779 study that combination therapy is required for significant clinical impact. The challenge, of course, lies in choosing the most promising second drug from an almost infinite number of possibilities. Based on earlier work from us and others, combined EGFR/mTOR blockade is one logical choice, because PTEN loss predicts for resistance to EGFR inhibitors in patients with the mutant EGFRviii variant [18,46–48]. Another possibility is combined PI3K/mTOR blockade to prevent rapamyin-induced activation of Akt caused by loss of negative feedback [27,28]. The time-to-progression analysis in our study suggests that the prognosis of these patients is worse, therefore inhibitors that act upstream of Akt may be useful to prevent this complication. Indeed, one dual PI3K/mTOR inhibitor has shown superiority to a pure mTOR inhibitor in preclinical models [49].

Although the findings reported here are directly relevant to mTOR inhibitors in glioblastoma, the implication is that these drugs will have activity in a broad range of cancers with PI3K/Akt pathway dysregulation—through PTEN loss, PI3K p110α mutation, AKT gene amplification, or other mechanisms. Recently, mTOR inhibitors have shown clinical activity in metastatic kidney cancer, where the frequency of PTEN loss is low [50]. The molecular basis for sensitivity in this disease is unknown, but loss of the von Hippel-Lindau (VHL) tumor suppressor and subsequent mTOR-dependent HIF-1α expression is one postulated mechanism [51]. For reasons similar to those articulated above for glioblastoma, mTOR-based combination therapies are also under consideration in kidney cancer. The neoadjuvant clinical trial design described here should be easily exportable to other cancers in which experimental drug delivery can be timed prior to a planned surgical excision of tumor, and such an approach is consistent with recent national efforts to speed clinical development through novel trial designs [52].

## Supporting Information

Figure S1 Digital Scoring of Immunohistochemical Stains Adjacent tissue sections from each tumor were stained with antibodies against Ki-67, phospho S6 ribosomal protein (S6RP), phospho-PRAS40, and PTEN (unpublished data). Five areas per slide, each representing approximately 500–1,000 tumor cells, were selected for digital scoring. Image conversion and scoring was performed using Soft Imaging System Software. The distribution of immunoreactivity within these 2,500–5,000 cells was graphed for each sample as a box plot. The “fold change” (F.C.) in S6 immunoreactivity (Table 2 and Figure S2) was calculated for each tumor as the ratio between median staining score in the S2 and the median staining score in the S1 sample.
(135 KB PDF)

Figure S2 S6 Phosphorylation at Ser 235/236 in Matched S1/S2 Tumor Tissue Pairs (A) Representative IHC staining results for pSer 235/236 S6. Shown are examples for a tumor with biochemical mTOR inhibitor resistance (patient 2) compared to a tumor with marked mTOR inhibition in response to rapamycin (patient 8).
(B and C) Quantification of S6 phosphorylation at Ser 235/236 in matched S1/S2 tumor samples from 14 patients in the rapamycin clinical trial cohort (B) and nine glioblastoma patients who did not receive rapamycin prior to S2 (C). For additional information regarding IHC scoring methodology, see Figure S1 and Text S1.
(1.2 MB PPT)

Figure S3 S6 Phosphorylation at Ser 240/244 in Matched S1/S2 Tumor Tissue Pairs IHC-based quantification of S6 phosphorylation at Ser 240/244 in (A) matched S1/S2 tumor samples from 14 patients in the rapamycin clinical trial cohort and (B) matched S1/S2 tumor samples from nine glioblastoma patients who did not receive rapamycin prior to S2.
(206 KB PPT)

Figure S4 CD31 Immunostaining of Representative Tumor Tissue Sections from Rapamycin-Treated Tumors with Low (Patients 1 and 3) Versus High (Patients 5 and 15) Intratumoral Rapamycin Concentrations Arrows indicate CD31 IHC-positive intratumoral vasculature.
(159 KB PDF)

Figure S5 Frequency of Genomic Aberrations in S2 Samples by Array-CGH Genomic DNA microdissected from fresh frozen tumor samples (S2) was subjected to oligonucleotide microarray-based CGH (aCGH) analysis. Aberrations were scored using CGH Analytics Software (Agilent) using the ADM1 algorithm (parameters listed in Text S1), and filtered to retain only aberrations with a log_2_ ratio less than −0.3 or greater than 0.3. The frequency of DNA copy-number increases (red, right of the zero axis for each chromosome) and losses (green, left of the axis) within the genome for the samples profiled is plotted in terms of percentage of the samples analyzed.
(56 KB PDF)

Figure S6 Gene Copy-Number Alterations Stratified by pPRAS40 Response CGH data for biopsies following rapamycin treatment were plotted using Cluster and TreeView Software (available at http://rana.lbl.gov/EisenSoftware.htm). DNA copy-number losses are represented in green, while gains are represented in red. Patient samples are stratified by the presence or absence of significant induction of pPRAS40 following rapamycin therapy as determined by IHC (see Figure 4B and Table 2).
(53 KB PDF)

Figure S7 IHC Scoring of PTEN Expression in Clinical Tumor Samples from Rapamycin Study Patients Compared to a Group of 44 Archival Glioblastoma Tissue Samples (A) Comparison of two PTEN IHC scoring methods (*x*-axis: manual score; *y*-axis: image-guided digital score) in 44 archival glioblastoma samples. The manual score compares PTEN expression in tumor cells to PTEN expression in adjacent endothelial cells and assigns scores of 0 (absent in tumor cells), 1 (reduced in tumor cells relative to endothelial cells), and 2 (tumor cells staining similar to endothelial cells). Digital PTEN scores are based on absolute values of PTEN immunoreactivity as determined through an image-guided software scoring system (Figure S1). Digital PTEN scores were determined by an independent reviewer without knowledge of the manual PTEN score.
(B) Mean digital PTEN IHC score of tumors from the rapamycin study group before (S1) and during (S2) rapamycin therapy.
(18 KB PDF)

Figure S8 Radiographic Response to Rapamycin in 2/15 Study Patients Magnetic resonance imaging results for two patients who experienced a clinical response to single-agent rapamycin.
(A) Patient 8 received an extended preoperative course of rapamycin (25 d) due to an intercurrent upper respiratory infection and experienced >50% tumor regression by magnetic resonance imaging.
(B) Patient 11 showed radiographically stable disease for >16 mo postoperatively and died without evidence for tumor recurrence.
(72 KB PDF)

Table S1 Rapamycin-Related Adverse Events (11 KB PDF)

Table S2 *PTEN* Copy Number (Log_2_ Ratio) and Missense Mutations in Tumor Tissue Genomic DNA was extracted from microdissected tumor cells. N/A: no fresh frozen tumor aliquot available for analysis. Log_2_ ratios ≤ −0.3 are consistent with DNA copy-number loss.
(13 KB PDF)

Text S1 Supplementary Methods (42 KB DOC)

Text S2 Protocol (254 KB DOC)

Text S3 CONSORT Checklist (59 KB DOC)

## Abbreviations

CGH: comparative genomic hybridization
GBM: glioblastoma
IHC: immunohistochemistry
KPS: Karnofsky performance score
mTOR: mammalian target of rapamycin
PTEN: phosphatase and tensin homolog deleted on Chromosome 10
S1: surgery 1
S2: surgery 2
TTP: time to progression
