# Supplementary material for: Antitumor Activity of Rapamycin in a Phase I Trial for Patients with Recurrent PTEN-Deficient Glioblastoma
Source: PLoS Med. 2008 Jan 22;5(1):e8. doi: 10.1371/journal.pmed.0050008 (PMC2211560; doi:10.1371/journal.pmed.0050008)
Supplement: Supplementary file 2 [file pmed.0050008.sg004.pdf]

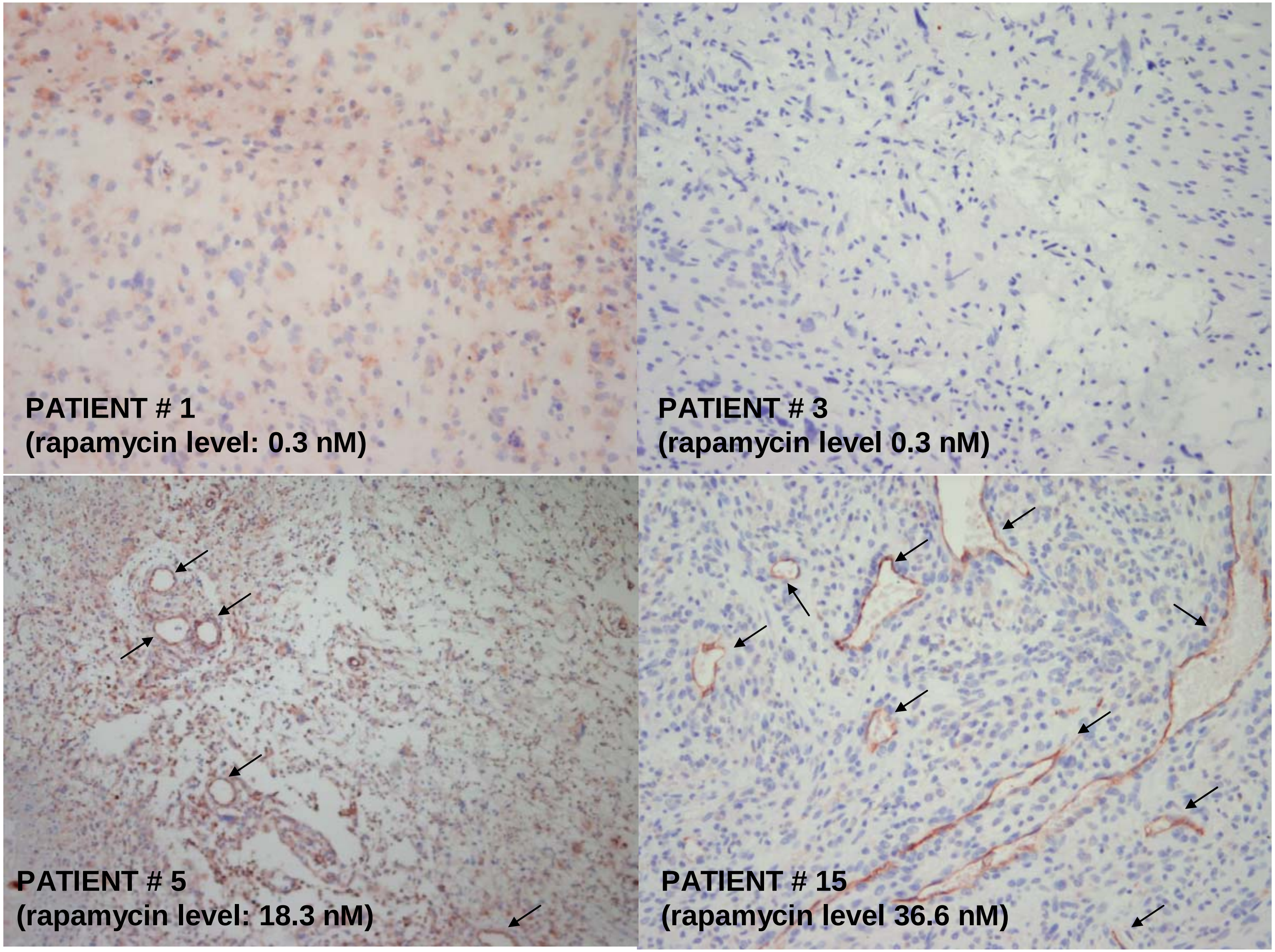
PATIENT # 1
(rapamycin level: 0.3 nM)
PATIENT # 3
(rapamycin level 0.3 nM)
PATIENT # 5
(rapamycin level: 18.3 nM)
PATIENT # 15
(rapamycin level 36.6 nM)
