# Supplementary material for: Antitumor Activity of Rapamycin in a Phase I Trial for Patients with Recurrent PTEN-Deficient Glioblastoma
Source: PLoS Med. 2008 Jan 22;5(1):e8. doi: 10.1371/journal.pmed.0050008 (PMC2211560; doi:10.1371/journal.pmed.0050008)
Supplement: Supplementary file 4 [file pmed.0050008.sg006.pdf]

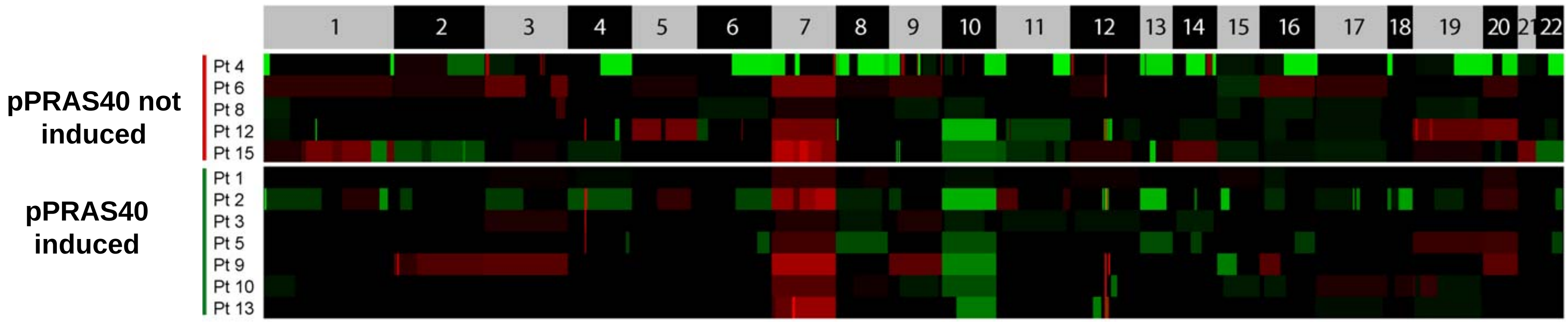
pPRAS40 not induced
pPRAS40 induced
Pt 4
Pt 6
Pt 8
Pt 12
Pt 15
Pt 1
Pt 2
Pt 3
Pt 5
Pt 9
Pt 10
Pt 13
1
2
3
4
5
6
7
8
9
10
11
12
13
14
15
16
17
18
19
20
21
22
