# Supplementary material for: Antitumor Activity of Rapamycin in a Phase I Trial for Patients with Recurrent PTEN-Deficient Glioblastoma
Source: PLoS Med. 2008 Jan 22;5(1):e8. doi: 10.1371/journal.pmed.0050008 (PMC2211560; doi:10.1371/journal.pmed.0050008)
Supplement: Supplementary file 5 [file pmed.0050008.sg007.pdf]

A.)

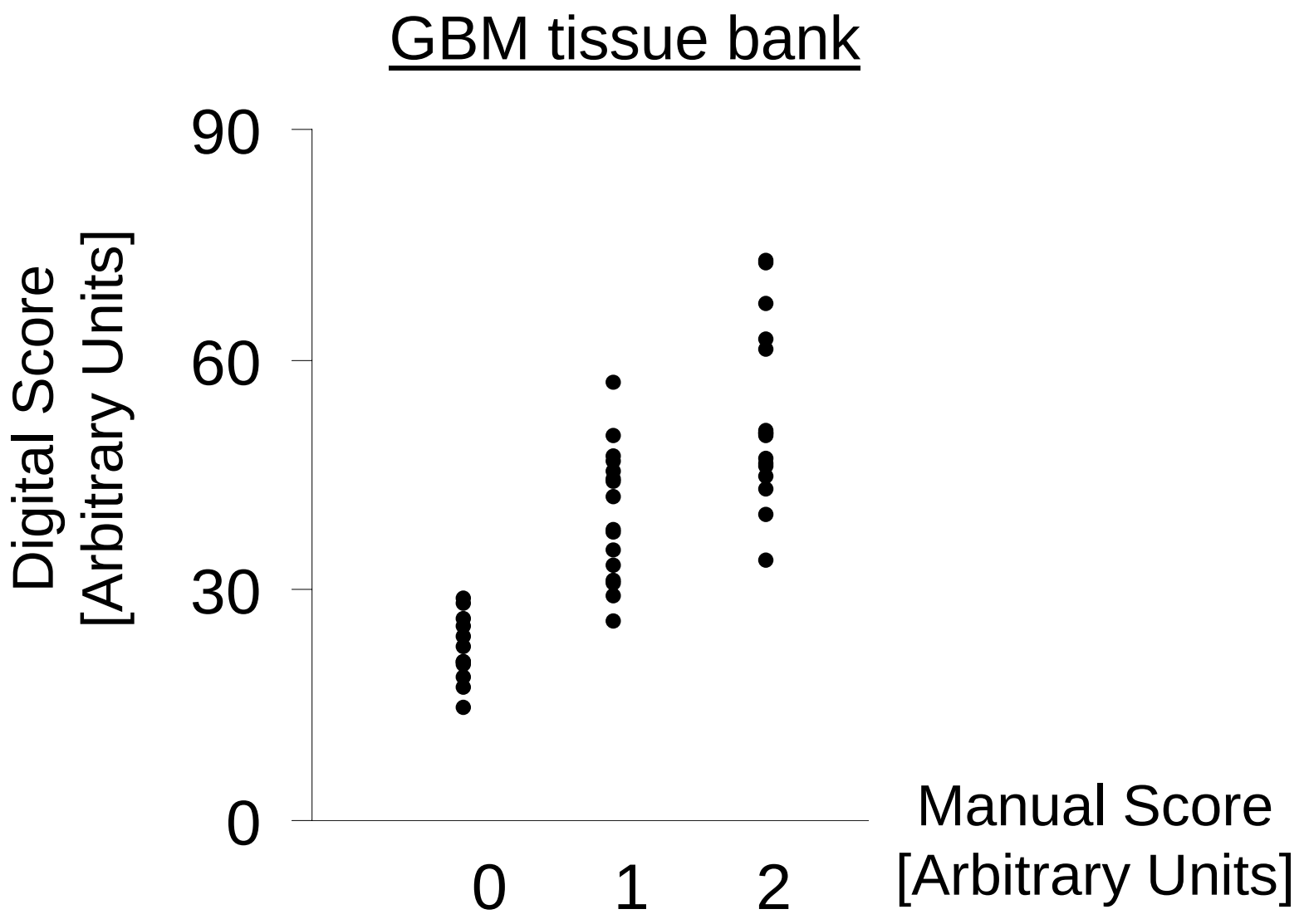

PTEN manual score (number of samples)	PTEN digital score	
	mean	range
0 (n = 12)	21.7	14.4 – 28.9
1 (n = 16)	39.8	25.7 – 56.8
2 (n = 16)	50.1	33.8 – 72.9

B.)

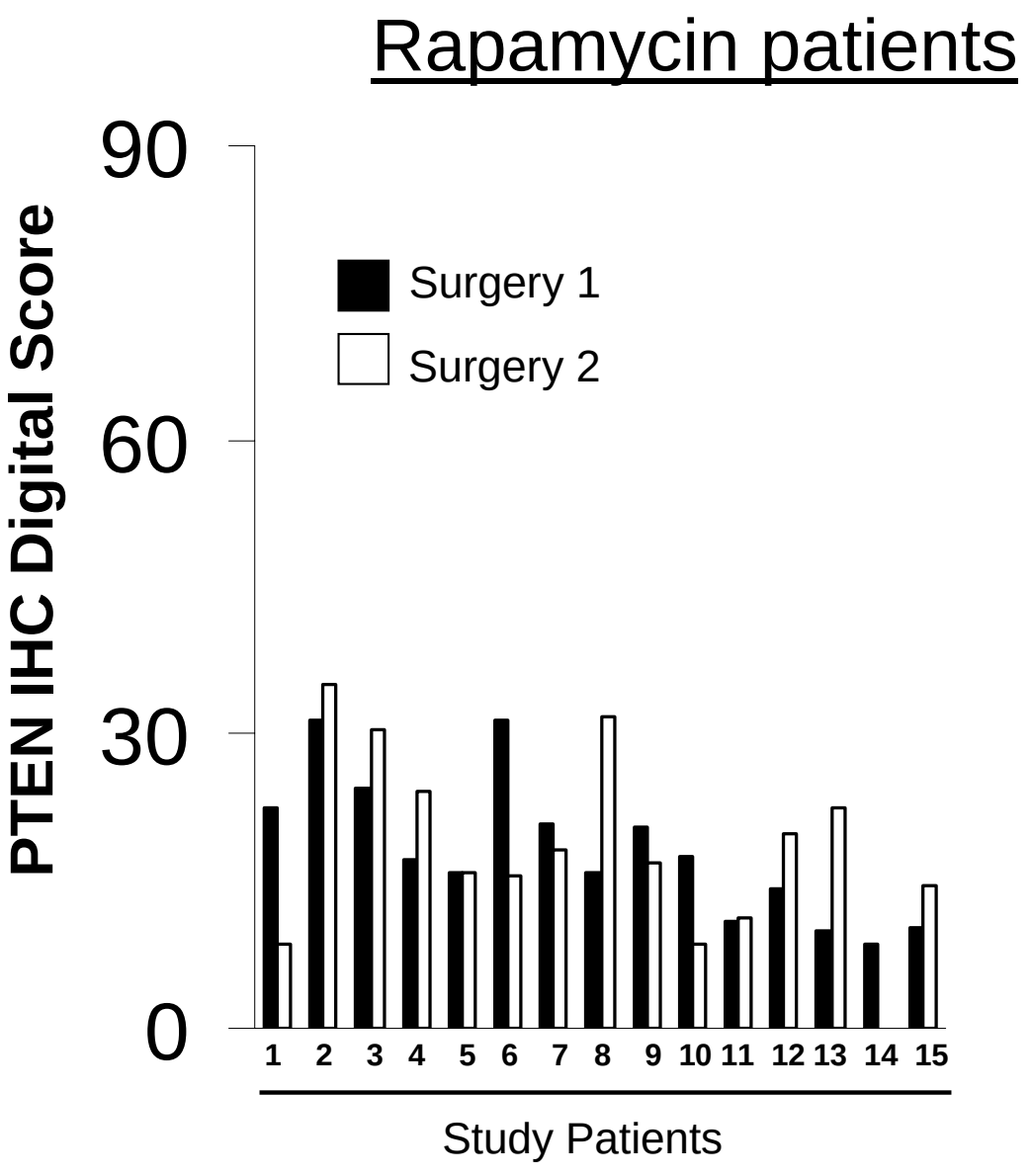

Surgery 1 (mean ± SD): 18.1 +/- 7.2
Surgery 2 (mean ± SD): 19.5 +/- 8.4
