# Supplementary material for: Antitumor Activity of Rapamycin in a Phase I Trial for Patients with Recurrent PTEN-Deficient Glioblastoma
Source: PLoS Med. 2008 Jan 22;5(1):e8. doi: 10.1371/journal.pmed.0050008 (PMC2211560; doi:10.1371/journal.pmed.0050008)
Supplement: Supplementary file 6 [file pmed.0050008.sg008.pdf]

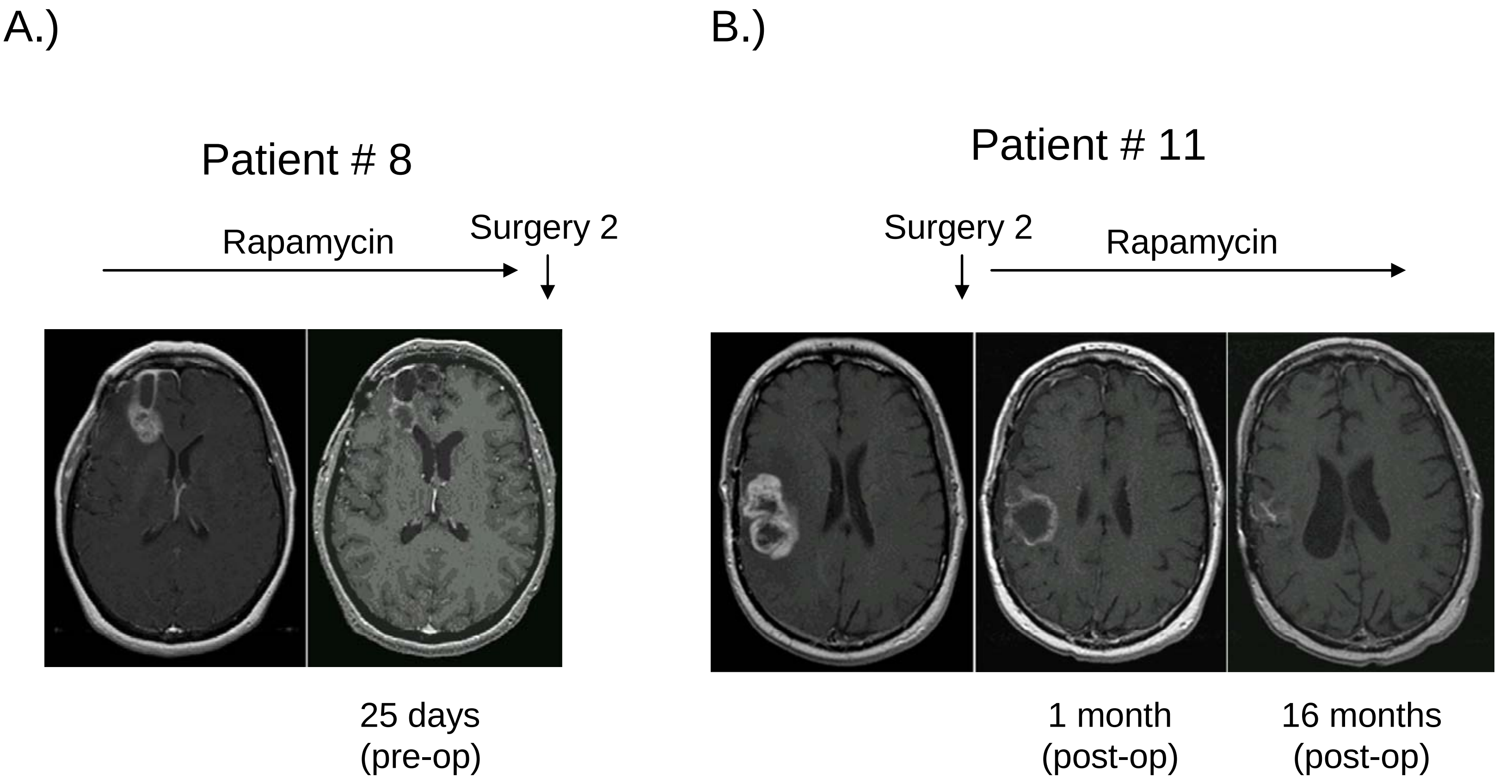

A.)
Patient # 8
Rapamycin
Surgery 2
25 days
(pre-op)
B.)
Patient # 11
Surgery 2
Rapamycin
1 month
(post-op)
16 months
(post-op)
