# Supplementary material for: Antitumor Activity of Rapamycin in a Phase I Trial for Patients with Recurrent PTEN-Deficient Glioblastoma
Source: PLoS Med. 2008 Jan 22;5(1):e8. doi: 10.1371/journal.pmed.0050008 (PMC2211560; doi:10.1371/journal.pmed.0050008)
Supplement: Supplementary file 7 [file pmed.0050008.st001.pdf]

Supplementary Table 1

Patient Number	Rapamycin Dose [mg]	Grade 3 Adverse Events*
1	2	N/A
2	2	N/A
3	2	N/A
4	5	N/A
5	5	N/A
6	5	N/A
7	5	Hypokalemia
8	5	Hypercholesterolemia, Hypokalemia
9	10	Hypokalemia
10	10	Leukopenia
11	10	Thrombocytopenia, Leukopenia
12	10	N/A
13	10	N/A
14	10	N/A
15	10	N/A

* No Grade 4 Adverse Events were observed
