# Supplementary material for: Antitumor Activity of Rapamycin in a Phase I Trial for Patients with Recurrent PTEN-Deficient Glioblastoma
Source: PLoS Med. 2008 Jan 22;5(1):e8. doi: 10.1371/journal.pmed.0050008 (PMC2211560; doi:10.1371/journal.pmed.0050008)
Supplement: Supplementary file 8 [file pmed.0050008.st002.pdf]

Supplementary Table 2

Patient Number	Surgery 1	Surgery 2	
	PTEN log 2 ratio (array cGH)	PTEN log 2 ratio (array cGH)	PTEN missense mutations
1	-0.7	-0.1	none detected
2	-0.7	-0.7	none detected
3	-0.5	-0.1	none detected
4	N/A	0.0	none detected
5	N/A	-0.3	none detected
6	N/A	0.0	none detected
7	N/A	N/A	**H61Y**
8	N/A	-0.1	none detected
9	N/A	-0.5	**R173C**
10	N/A	-1.2	none detected
11	N/A	N/A	N/A
12	-0.8	-0.7	none detected
13	N/A	-0.5	**V217F**
14	N/A	N/A	N/A
15	N/A	-0.4	none detected
**mean**		-0.4	
**median**		-0.3	
