# Supplementary material for: Antitumor Activity of Rapamycin in a Phase I Trial for Patients with Recurrent PTEN-Deficient Glioblastoma
Source: PLoS Med. 2008 Jan 22;5(1):e8. doi: 10.1371/journal.pmed.0050008 (PMC2211560; doi:10.1371/journal.pmed.0050008)
Supplement: Supplementary file 9 [file pmed.0050008.sd001.doc]

**SUPPLEMENTARY METHODS**

**Rapamycin dosing and drug safety monitoring:** Patients were assigned to three rapamycin dose cohorts. The first three patients were enrolled at the initial dose level of 2 mg per day. After a four week evaluation period for dose limiting toxicity, a minimum of 3 more patients could be enrolled at the next dose level. Rapamycin was given as an oral preparation once daily with an initial loading three times the daily maintenance dose (e.g., 6 mg for 2 mg dose cohort). Presurgical dosing included the loading dose followed by the daily maintenance dose until surgery. After the patient recovered from the effects of surgery, identical loading and maintenance doses (e.g., 6 mg and 2 mg, respectively) were administered. Patients remained on treatment with daily rapamycin until they developed disease progression as defined by ≥ 25% increase in all measurable lesions on MRIs, appearance of any new lesion/site, or death. Assessment of drug safety was done following a routine schedule and supplemented by additional evaluation whenever a patient reported a new symptom. Routine monitoring included complete blood counts (weekly), peripheral blood rapamycin levels (weekly), serum chemistry profiles (every 4 weeks), complete physical examination (every 4 weeks), and brain magnetic resonance imaging (every 8 weeks). Toxicities were graded according to the National Cancer Institute Common Toxicity Criteria version 2.0. Treatments were held until recovery to ≤ grade 1 toxicity for ANC < 1000/µl, platelets < 80,000/ µl, grade 3 or grade 4 non-hematologic toxicities, and grade 4 hypercholesterolemia or hypertryglyceridemia. Rapamycin was resumed at a 25 % dose reduction for ANC 750-1000/µl, platelets 50,000-80,000/µl, and a grade 3 non-hematologic AE. A 50 % dose reduction was made for ANC < 750/µl, platelets < 50,000/µl, or grade 4 non-hematological AE. Patients were allowed two dose reductions. Lipid lowering agents were initiated for hypercholesterolemia or hypertriglyceridemia. Rapamycin-related adverse events (AEs) were defined as events not present at baseline or present at baseline but worsened during treatment. Overall, five patients experienced rapamycin-related grade 3 adverse events *(Suppl. Table 1)*. Patient 11 with longterm corticosteroid dependency developed grade 1 dyspnea about 3 months after starting Rapamycin and received antifungal therapy for presumed coccidiomycosis. This patient died 538 days after resuming rapamycin of pulmonary failure and a brain MRI obtained one month prior to death *(Suppl. Fig. 3)* showed no evidence for glioblastoma recurrence.

**Scoring of immunohistochemical stains.** Immunohistochemical screening of tumor samples for PTEN expression was based on a manual scoring method which compares PTEN staining of tumor cells to the PTEN staining of endothelial cells within the same viewfield and assigns scores of 0 (absent in tumor cells), 1 (reduced in tumor cells relative to endothelial cells), and 2 (tumor cells staining similar to endothelial cells) [19,20]. Digital scores for PTEN, phospho-S6, and phospho-PRAS40 are based on absolute staining intensity of tumor cells as quantified following false-color conversion. PTEN, phospho-S6, phospho-PRAS40, and Ki-67 were scored in representative and matching areas of consecutive tissue sections *(Suppl. Fig. 1),* excluding areas with large vessels and/or prominent reactive changes (e.g., reactive astrogliosis or lymphocytic infiltration). Each tumor core image was scanned at 20 X magnification using a colorview II camera mounted on an Olympus BX61 microscope and borders between individual cells were approximated using a separator function of the Soft Imaging Software (with the parameter of Smooth and Fine/Coarse, 2 and 10 respectively). Quantitative analysis was done using HSI color algorithm based on hue, saturation and intensity. Saturations of the separated cell in the images were quantified in the red-brown hue range to exclude the negative staining area with hematoxylin nuclear staining. To compare the staining intensity of all slides, mean saturation of total cells on each image was quantified and calculated. For Ki-67 staining scoring, separated cells were quantified with red brown hue range (positive staining) and total hue range (all cells) after cell border separation and Ki-67 labeling index was calculated based on these numbers. For p-PRAS40 scoring, mean saturation of the positive staining tumor cells was quantified in the red-brown hue range, followed by multiplying the positive cell ratio in the same image to evaluate both staining intensity and staining distribution. pS473 Akt was quantified on a scale of 0-2; 0^+^ = no staining, 1^+^ = mild intensity cytoplasmic staining, and 2^+^ = strong cytoplasmic staining. Staining of 1^+^ and 2^+^ were considered positive. Significant nuclear p-AKT staining was not observed in these samples, consistent with our previous work .

**Comparative Genomic Hybridization.** 800 ng tumor DNA or normal male control DNA (Promega, Madison, Wisconsin) was digested with 1 uL AluI (10U/uL; Invitrogen, Carlsbad, CA) and 1 uL Rsa I (10U/uL; Invitrogen) in 13.33 uL total volume at 37C for 2 hours, followed by heat inactivation at 65C for 10 minutes. Digestion was subsequently verified via agarose gel. DNA samples were labeled using the Bioprime CGH kit (Invitrogen): 13 uL of each digestion was combined with 20 uL 2.5X random primers and incubated at 99C for 5 minutes. Reactions were subsequently snap-cooled on ice. 5 uL Cy5-dUTP (tumor) or Cy3-dUTP (control) (GE Healthcare) was added along with 1 uL Exo-Klenow; reactions were mixed an incubated at 37C for 2 hours. Reactions were stopped with 5 uL stop buffer and cleaned over a Vivaspin 500 column (Sartorious Goettingen, Germany). Cy5 and Cy3 reactions were combined and hybridized in a total volume of 500 uL with 50 uL human Cot-1 DNA (1 mg/mL; Invitrogen), 25 uL yeast tRNA (5 ug/uL; Sigma-Aldrich, St. Louis, Missouri), 50 uL 10X Control Targets (Agilent), and 250 uL 2X Hybridization Buffer (Agilent). Probe mixtures were denatured via boiling 3 minutes, followed by pre-incubation at 37C for 30 minutes. Probes were added to arrays and arrays were hybridized in a rotating oven (Agilent) at 65C for 48 hours at 10 RPM. Arrays were washed for 5 minutes in CGH Wash Buffer 1 (0.5X SSPE, 0.005% N-Lauroylsarcosine) at room temperature, 12 minutes in CGH Wash 2 (0.1X SSPE, 0.005% N-Lauroylsarcosine) at 37C, and 1 minute in Stabilization and Drying Solution (Agilent) at room temperature.

Arrays were scanned using an Agilent DNA Microarray Scanner at 10 micron resolution. Data was extracted using Agilent Feature Extraction Software v9.1.1 using default CGH extraction parameters (Agilent Protocol CGH-v4_91). We excluded from consideration any samples for which a derivative log ratio spread was greater than 0.35. Data were visualized using CGH Analytics Software (Agilent) and data were further normalized using the data centralization protocol using a threshold value of 6.0 and a bin size of 10.

Aberration calling was performed using CGH Analytics using the ADM1 algorithm. For the purposes of PTEN log2 ratio calculation, an ADM1 threshold of 6 was used with no aberration filtering. Samples for which no significant copy-number change in PTEN was detected were assigned a log2 ratio of zero. For the purposes of calculating the frequency of aberration across the genome (Suppl. Fig. 4), we further filtered aberrations to retain only those spanning at least two CGH probes and with a log2 ratio less than -0.3 or greater than 0.3. Suppl. Fig. 5 was generated using Cluster and TreeView software (available at <http://rana.lbl.gov/EisenSoftware.htm>). Aberration called data (ADM1, threshold =6) exported from CGH Analytics was used. Aberrations representing >2-fold loss or gain are represented in bright green and red, respectively.
