# Supplementary material for: Antitumor Activity of Rapamycin in a Phase I Trial for Patients with Recurrent PTEN-Deficient Glioblastoma
Source: PLoS Med. 2008 Jan 22;5(1):e8. doi: 10.1371/journal.pmed.0050008 (PMC2211560; doi:10.1371/journal.pmed.0050008)
Supplement: Supplementary file 10 [file pmed.0050008.sd002.doc]

# UCLA - Neuro-oncology Program

A modified Phase I/II Trial of rapamycin in Patients with Glioblastoma Multiforme

Page

1.0	OBJECTIVES	2

2.0	BACKGROUND	2

3.0	DRUG INFORMATION	6

4.0	ELIGIBILITY CRITERIA	11

5.0	STRATIFICATION/DESCRIPTIVE FACTORS	12

6.0	TREATMENT PLAN	12

7.0	PRETREATMENT EVALUATION	16

8.0	EVALUATION DURING STUDY	17

9.0	CRITERIA FOR EVALUATION AND ENDPOINT DEFINITIONS	19

10.0	CRITERIA FOR REMOVAL FROM STUDY	21

11.0 STATISTICAL CONSIDERATIONS	21
12.0 DISCIPLINE REVIEW	23
13.0 ANALYTICAL ASSAY	23
14.0 LABORATORY CORRELATES	23
15.0 DATA SAFETY MONITORING BOARD	23
16.0 REFERENCES	25
17.0 APPENDIX	26

**PARTICIPANTS**

| **PRINCIPAL INVESTIGATORS**  Timothy Cloughesy, MD  710 Westwood Plaza, Suite 1-230  Los Angeles, CA 90095 | **AGENTS**  **1. Rapamycin** |
| --- | --- |
| **Co- INVESTIGATORS**  Charles Sawyers  Paul Mischel  Linda Liau  Johannes Czernin   | **BIOSTATISTICIAN**  Robert Elashoff |

**1.0	OBJECTIVES**

1.1 **Modified Phase I**
    1.1.1 To establish effective dose for rapamycin as determined by inhibition of S6 kinase and determination of tissue and blood drug level.
    1.1.2 To define the safety profile of daily rapamycin in patients with glioma.
1.2 **Phase II**
    1.2.1 To determine the efficacy of rapamycin in PTEN null patients not on enzyme inducing anticonvulsants with recurrent malignant glioma as measured by 6 month-progression free survival and objective response.
    1.2.2 To further evaluate the safety profile of rapamycin in patients with malignant glioma not on enzyme inducing anticonvulsants.

**2.0	BACKGROUND**

Despite progress made using combination therapies including surgery, radiation and/or chemotherapy, the treatment of malignant gliomas remains problematic with a typical median survival of 10 months for patients with newly diagnosed glioblastoma multiforme and 24-36 months for patients with anaplastic astrocytoma . The time to tumor progression of patients with recurrent malignant gliomas is eight weeks and the median survival for adults with recurrent glioblastoma multiforme (GBM) is 25 weeks and 47 weeks for recurrent anaplastic astrocytoma (MD Anderson Cancer Center historical data). Chemotherapy treatment for malignant glioma has limitations given the low activity of available antineoplastic agents, compromised delivery of these agents to at least partially privileged intracranial sites, the emergence or de-novo presence of resistance to these agents, and the sensitivity of the brain to irreversible damage from any therapeutic modality. Effective agents with novel mechanisms of action need to be evaluated in malignant glioma. As these novel agents move into the clinical setting many of them show the ability to block specific focal molecular pathways. As these pathways are better defined it will be critical to design clinical trials that develop molecular inclusion criteria. This will be critical as likely only a percentage of tumors will exhibit activation of these focal molecular pathways and maybe a smaller fraction will be strongly dependent upon that pathway for maintaining tumor growth.

**2.1.	Rapamycin:**

Sirolimus (Rapamycin, Rapamune®), is a macrolide originally isolated from *Streptomyces hygroscopicus.* Rapamycin causes cell cycle arrest at G1 via inhibition of key signaling pathways, via inhibitions of RNA translation. Its ester analog is CCI-779 which has similar if not identical biologic effects. It is a potent immunosuppressant and like other natural immunosuppressants such as cyclosporin A and FK-506, interacts with signal transduction systems that operate in both normal T cells and tumor cells (9, 12). These agents bind to a ubiquitous family of proteins known as immunophilins, inhibiting their enzymatic activity. The immunophilins are subdivided into cyclophilins and FK-506 binding proteins (FKBP). Rapamycin binds to FKBP-12 to form a complex that interacts with a novel kinase, termed mammalian target of rapamycin (mTOR, also known as FRAP or RAFT), blocking its activity (1-6). This, in turn, results in inhibition of key signal transduction pathways, including those regulated by p70s6 kinase and by phosphorylation of the eukaryotic initiation factor (elF)-4E-binding protein, PHAS-I (phosphorylated heat – and acid-stable protein) (13-14). Inhibition of these key-signaling pathways results in inefficient translation of mRNAs important for cell-cycle progression through G1 phase (8, 16).

2.2. **Pre Clinical Activity of Rapamycin:**

Because rapamycin is a potent and highly selective inhibitor of the kinase mTOR, it should be considered as a signal transduction inhibitor. Although originally developed to inhibit mTOR in normal T-cells in order to induce immune suppression, it is now clear that the mTOR kinase plays a role in the growth and survival of tumor cells. As expected from such a model, rapamycin has antitumor activity against a range of tumor cell lines derived from different types of cancer, including glioblastoma. These results have provided the rationale for the development of rapamycin and rapamycin analogues such as CCI-779 for the treatment of various cancers, as outlined below.

Recent preclinical studies performed at UCLA (and confirmed by investigators elsewhere) has provided new insight into the molecular basis of the therapeutic response to rapamycin. These studies demonstrated that tumor cell lines lacking the tumor suppressor gene PTEN are exquisitely sensitive to rapamycin and the analogue CCI-779. The molecular basis for this enhanced sensitivity is due to upregulation of a biochemical signal transduction pathway involving PTEN and mTOR. Furthermore, the responses to rapamycin observed in the preclinical models occurred at does that inhibit mTOR and correlated with biochemical inhibition of mTOR (Neshat et al, PNAS, 2001; Podsypanina et al, PNAS, 2001).

The collective preclinical data from studies of rapamycin and the analogue CCI-779 establish the following conclusions.

1) Rapamyin and CCI-779 have anti-cancer activity against cell lines from a broad range of tumor types.
2) The therapeutic response correlates with inhibition of the mTOR kinase.
3) Cell lines and tumors from genetically modified knockout mice which have lost the PTEN tumor suppressor are uniformly sensitive to rapamycin.

These preclinical data predict that rapamycin should have clinical activity against tumors which lack PTEN when administered at doses that inhibit mTOR. This clinical protocol will test this hypothesis in patients with recurrent glioblastoma whose tumors have deletions or mutations in PTEN. This determination will be made using immunohistochemical assays developed at UCLA. Rapamycin will be administered at doses sufficient to inhibit mTOR, which are essentially identical to the doses currently administered to kidney transplant patients that produce immune suppression. In the first phase of the protocol, inhibition of mTOR in the tumor cells will be confirmed using additional immunohistochemical assays developed at UCLA.

**2.3.	Rationale for studies on tumor specimens:**

Recent preclinical studies performed at UCLA indicate that tumor cell lines lacking PTEN have enhanced sensitivity to rapamycin and its analogue CCI-779 (Neshat et al PNAS, 2001). Further preclinical studies at UCLA using low passage patient-derived glioblastoma cell cultures have shown that low PTEN protein expression is strongly correlated with sensitivity to rapamycin/CCI-779 mediated growth arrest (Smith et al., AACR 2002). These studies suggest that rapamycin may be an effective targeted molecular therapy for patients with PTEN-deficient Glioblastoma Multiforme. To detect PTEN status on paraffin-embedded biopsy samples, we have recently optimized an an immunohistochemical assay. Since all patients will be enrolled at recurrence, the original paraffin-embedded biopsy material will be available for PTEN immunohistochemical analysis.

**2.4	Trial Design:**

Based on the preclinical and clinical data outlined above, we propose a modified Phase I/II study of **rapamycin** in patients with malignant glioma. There is increasing evidence that brain tumor patients receiving enzyme-inducing anti-epileptic drugs (EIAEDS) have markedly altered pharmacokinetics, resulting in accelerated drug metabolism. This may result in decreased levels of certain chemotherapeutic agents when administered at conventional doses. Failure to achieve adequate plasma levels of such agents may account for the lack of efficacy in past brain tumor trials. **Rapamycin** is a substrate for the cytochrome P450 isoenzyme 3A4. It is likely that the patients taking EIAEDS will have increased drug metabolism and a higher MTD than patients who are not taking EIAEDS. Additionally when MTD is reached in the patient group on EIAEDs pharmacokinetics are not equivalent to the patients not on EIAED. Therefore there may not be a way to provide equivalent doses of Drug to those using EIAED. With the advent of newer none enzyme inducing anticonvulsants, patients can be safely treated with monotherapy of these agents. Therefore we will only be enrolling patients who are not on EIAEDs in order to ensure comparable pharmacokinetic profiles.

To determine if Rapamycin at our selected dose has activity *in vivo,* a Phase Ib study will be performed on tumor specimens obtained from patients undergoing resection for recurrent tumor. The concentrations of rapamycin will be determined. Post treatment biopsies for various assays of some known biological targets of activity of Rampamycin will be performed. When possible, the patient’s tumor specimen at diagnosis will be obtained for comparison and if patients on therapy undergo any surgical intervention, this tissue will also be assayed. It is hoped that these studies will provide information concerning the concentration of drug achieved in glioma specimens and whether there is *in vivo* evidence of inhibition of these key signaling pathways. Phase II dose will be based upon the success of inhibition of these key signaling pathways. Blood levels will also be obtained during the phase I study to aide in adjusting dose for the phase II study

Our predictions with regard to tumor specimen studies are as follows:

1) Inhibition of mTOR by rapamycin will cause:
   a. Low levels of activated SK6 and 4E-BP1

For this trial we will be using a dosing regimen similar to that used to treat Transplant patients who require immune suppression to prevent graft vs. host reactions. Please see the package insert and section 3 regarding details of pharmacokinetics, preclinical and clinical data of rapamycin. Other details regarding experience with CCI-779 is below. It should be noted that the dosing paradigm with CCI-779 is quite different from the proposed study.

**2.5	Pre-clinical pharmacology of CCI-779:**

Pre-clinical pharmacokinetic studies of CCI-779 indicated that drug exposure was dose-dependent, but less than proportional in monkeys and rats that received doses of 0.1-2.5 mg/kg. The elimination half life was approximately 8-16 hours and the volume of distribution (Vdss) was large in both rats (13-19 L/kg) and monkeys (1-5 L/kg)- the Vdss of rapamycin was 1.1 to 1.3 L/kg after IV administration in humans and the half-life is approximately 40-60 hours.

Minimal bio-transformation products were observed in blood and plasma of rats. In human liver microsomal fractions, the drug was primarily metabolized by cytochrome P450 3A (CYP3A). Major metabolites include desmethyl-rapamycin, desmethyl-CCI-779, hydroxy-rapamycin and hydroxy-CCI-779. Non-CYP bio-transformation results in the formation of rapamycin, seco-CCI-779, and seco-rapamycin. Since extensive RBC partitioning has been reported with rapamycin, this is expected to occur with CCI-779 as well. Greater potential for partitioning into RBCs occurs in monkeys and humans than in rats.

**2.6	Pre-clinical Toxicology:**

Pre-clinical toxicology studies were performed in rats and monkeys. The drug was administered daily for 14 days or daily for 5 days, intravenously (IV), every 2 weeks for a total of 4 courses. No life-threatening toxicity was observed. Thymic and/or lymphoid atrophy, increased red blood cell (RBC) counts and decreased white blood cell (WBC) and platelet counts occurred. Other toxicities included pancreatic islet cell vacuolation with associated hyperglycemia and cataracts in rats; testicular tubular degeneration and hypospermia in rats and monkeys; and decreased ovarian weights in rats. All of these adverse effects have been observed in studies of rapamycin, cyclosporin A and /or FK-506. CCI-779 also caused other toxicities that have been reported with rapamycin. Myocardial degeneration in rats, which is thought to be related to activation of latent rodent parvovirus, occurred, but there were no effects on cardiovascular function in rats or monkeys. Colitis and chronic diarrhea occurred in monkeys. Increased numbers of pulmonary alveolar macrophages were observed in rats. CCI-779 showed no mutagenic effects *in vitro* or *in vivo.*

**2.7	Clinical studies of CCI-779**

The majority of Cancer studies performed using rapamycin have been done using CCI-779. IT is not clear that the experience with CCI-779, with regard to response or toxicity, will be any different than the planned dosing scheme using rapamycin. The following includes experience of CCI-779 in clinical cancer studies. CCI-779 is currently being evaluated in 2 single agent Phase I studies in patients with solid tumors, a single agent Phase II study in patients with renal cancer and 2 ongoing Phase I studies of CCI-779 in combination with other chemotherapeutic agents (gemcitabine and 5-fluorouracil). Two schedules of administration are being studied in the single agent Phase I studies: daily x 5 every 2 weeks and a single dose weekly, each administered as a 30-minute infusion. Dose levels studied to date with the daily schedule range from 0.75 mg/m^2^ to 19.1 mg/m^2^ for the heavily pretreated patients and 24 mg/m^2^ for the minimally pretreated patients. It appears that 15mg/m^2^ daily x 5 every 2 weeks is the MTD for heavily pretreated patients (based on dose reductions and delays because of Grade 3 AST/ALT elevations, Grade 3 vomiting and fatigue, and grade 3 thrombocytopenia at a dose of 19.1 mg/m^2^) and 19.1 mg/m^2^ for minimally pretreated patients (based on the inability to give repetitive doses of 24 mg/m^2^ because of Grade 3 stomatitis and grade 2-3 thrombocytopenia). Doses studied to date with the weekly schedule range from 7.5 mg/m^2^ to 220 mg/m^2^ and the MTD has not yet been reached in this trial.

CCI-779 has been well tolerated. The most frequent adverse events of clinical importance include myelosuppression (thrombocytopenia, neutropenia and anemia), cutaneous toxicity (including acneiform lesions/folliculitis and maculopapular, erythematous lesions), stomatitis/mucous membrane disorder, asthenia, nausea/vomiting, diarrhea, hyperglycemia and hypertriglyceridemia. Other adverse events reported in >20% of patients in either Phase I single agent study, regardless of drug relationship, include headache, cough, rhinitis/pharyngitis, dyspnea, fever, hypercholesterolemia, hypocalcemia, anorexia, epistaxis, peripheral edema, weight loss, nail disorder, catheter infection, pain, and taste perversion, based on preliminary information. In addition, decreased testosterone with loss of libido has been reported in men receiving CCI-779. Most of these events have been mild to moderate in severity, however some patients had their course of treatment delayed and in some cases dose reductions were necessary, as a result of adverse events. Some patients with hypertriglyceridemia have been treated with gemfibrozil and have been able to continue on study.

Serious adverse events considered possibly related to single agent CCI-779 include “diffuse infiltrative pneumopathy” (interstitial pneumonitis), infection including pneumonia, fever, cutaneous eruptions, urticaria, stomatitis, bipolar disorder, renal insufficiency, nausea, vomiting and diarrhea (C.difficile), and bleeding with a possible interaction with warfarin. **Pertinent to the patient population in this study, there have been 2 patients with malignant glioma on CCI-779 who died from intratumoral hemorrhages. These patients were both on anticoagulants and the bleeding may have been related to CCI-779 (Wyeth-Ayerst correspondence). Another adverse event that may be related to CCI-779 was seen in a patient with metastatic renal cell cancer who developed pulmonary hypertension and heart failure. The patient had a prior history of hypertension and pericardial effusion.**

Acute hypersensitivity reactions (beginning shortly after the start of the IV infusion, usually but not always, with the first infusion, and ending shortly after the infusion) have been reported. Signs and symptoms have included flushing of the face and neck and in some cases involving the extremities and trunk, description of feeling hot, uncomfortable, and/or anxious, chest pain/tightness, shortness of breath, decrease in oxygen saturation and cyanosis, hypotension, lightheadedness, periorbital puffiness, nausea, back pain, numbness and tingling of hands/feet/face, and difficulty speaking. Some patients were able to receive subsequent infusions of CCI-779 following pre-medication with diphenhydramine with or without corticosteroids and/or a histamine H2-receptor antagonist. As of 4/2000, 3 patients receiving CCI-779 as a single agent in Phase I/II studies had to be discontinued from study because of drug-related adverse events: 2 cases for hypersensitivity reactions and one for nausea while on a lipid-lowering agent for hypertriglyceridemia. As of May 2000, 7 patients have died while on study or shortly after discontinuing from treatment, 6 from disease progression and one of gastrointestinal perforation and septic shock which was felt to be related to CCI-779/5-FU/leucovorin.

The most frequent laboratory changes of clinical importance include leukopenia/neutropenia, thrombocytopenia, anemia, increased serum cholesterol and trigylcerides, hyperglycemia, hypocalcemia, decreased serum testosterone, hypoproteinemia, increased AST/ALT and increased serum creatinine.

Preliminary evidence of antitumor activity has been reported with CCI-779 in Phase I trials. Tumor regression (generally less than 50%) has been reported in patients with several tumor types, including renal cell cancer, soft tissue sarcoma, cervical cancer, endometrial cancer, non-small cell cancer and non-Hodgkins lymphoma. Partial responses have been observed in renal cell and NSCL carcinomas

Preliminary pharmacokinetic data are available from patients at the early dose levels. Throughout the dose range of 7.5 to 80 mg/m^2^ (weekly schedule), CCI-779 exhibits generally increasing Cmax and AUC in whole blood with increasing dose. At lower doses (0.75 to 3.12 mg/m^2^ daily x 5 schedule), CCI-779 distributes preferentially into red blood cells, with a mean (range) blood/plasma (B/P) ratio of 9.8 (2.4-20). CCI-779 is extensively metabolized to rapamycin and other oxidative metabolites. With weekly dosing, mean clearance is 22 L/hour, volume of distribution at steady state is 306 L, and the half-life is 19.3 hours (range 13-39 hours). Measurable levels of rapamycin were also observed in blood. The mean exposure ratio (rapamycin to CCI-779) was roughly estimated to 3.2. Based on observed whole blood exposure (AUC) in mice and humans, a dose of 250 mg is comparable to or higher than effective doses observed in preclinical models for sensitive tumors.

**3.0	DRUG INFORMATION**

**3.1	Drug Name: Rapamycin (Rapamune, Sirolimus, AY-22989, NSC-226080, NSC-6006698)**

3.1.1 **Human Toxicity/ Adverse Events**

**Although Rapamycin at the typical dosing for transplant patients who require immunosuppression, has not been evaluated in glioma the following are toxicities that are seen in transplant patients who are treated with daily dosing at 2 or 5 mg daily. The following adverse events are provided from PDR:**

HEMATOLOGIC EFFECTS

SUMMARY: During clinical trials, ANEMIA was reported in 27% to 37% of patients receiving sirolimus.

THROMBOCYTOPENIA was also reported in 13% to 30% of patients during clinical trials (Prod Info Rapamune(R), 2001; MacDonald et al, 2001). Thrombocytopenia is an adverse effect of sirolimus therapy in renal transplant patients receiving therapy in renal transplant patients receiving cyclosporine/corticosteroids; it has also occurred during sirolimus monotherapy. Reductions in platelet count are dose-related and usually seen 9 or 10 days after initiation of treatment (Murgia et al, 1996; Groth et al, 1998; Kahan, 1998; Anon, 1999; MacDonald et al, 2001). With usual recommended doses (1 to 3mg/m(2) daily), thrombocytopenia was modest in one study (from 223,000/mm(3) at baseline to 209,000/mm(3)on day 15), whereas an additional decline of 60,000 cells/mm(3) was seen at this time with doses of 5 to 6 mg/m(2) daily (Murgia et al, 1996). Dose reduction or discontinuation of treatment is indicated based on the degree of thrombocytopenia. Normalization of platelet counts is seen within 2 weeks of discontinuation (Murgia et al, 1996).

LEUKOPENIA, which appears non-dose-related, occurs with sirolimus monotherapy or during combined use with cyclosporine in renal transplant patients. It is evident within 2 weeks of initiation of therapy and is evident within 2 weeks of initiation of therapy and is reversible upon discontinuation (Anon, 1999; Murgia et al, 1996; Groth et al, 1998; Slaton et al, 1995). Reductions in white count are usually modest, although counts below 4800/mm(3) were observed in 20% of patients by day 15 of treatment with 1 to 13 mg/m(2) daily in one trial (Murgia et al, 1996). Differential effects of the drug have not been presented. Sirolimus may impair erythroid recovery following transplantation of kidneys producing erythropoietin; this can usually be managed by therapy with erythropoietin or iron (Kahan, 1998).

CARDIOVASCULAR

CARDIOVASCULAR EFFECTS

During clinical trials, HYPERTENSION occurred in 43% to45% of patients receiving sirolimus 2 milligrams (mg)/day, and in 39% to 49% of patients receiving sirolimus 5 mg/day. Peripheral EDEMA occurred in 54% to 64% of patients receiving sirolimus (Prod Info Rapamune(R), 2001.

CAPILLARY LEAK SYNDROME was reported in 2 psoriasis patients receiving sirolimus. A female patient had received a single dose of sirolimus 8 milligrams (mg) /square meter (m(2)), followed in 1 week by consecutive daily doses at this same level. A male patient had been receiving 1 mg/m(2) daily for 1month. Both patients presented with symptoms of fever, hypotension, and lower extremity edema, after the third daily dose and after one month of daily sirolimus therapy in the female and male, respectively. Other symptoms reported were orthopnea, fatigue, dizziness, and weight gain. Laboratory analysis revealed normocytic anemia, negative direct/indirect Coombs tests and absence of serological evidence for hemolysis and parvovirus B-19. Following discontinuation of sirolimus, the female patient improved spontaneously within 72 hours, whereas the male patient required further treatment with intravenous furosemide and packed red blood cell transfusion. Neither patient reported sequelae at follow-up (Kaplan et al, 1999).

CENTRAL NERVOUS SYSTEM

CENTRAL NERVOUS SYSTEM EFFECTS

During clinical trials, HEADACHE occurred in 23% to 34%of patients treated with sirolimus; tremor (21% to31%) and insomnia (13% to 22%) were also reported. An association with sirolimus is difficult to determine for the latter adverse effect because patients were also receiving cyclosporine (Prod Info Rapamune(R), 2001).

ENDOCRINE/METABOLIC

LIPID ABNORMALITIES

SUMMARY: Hyperlipidemia (38% to 57%) and hypercholesterolemia (38% to 46%) are dose-related effects of sirolimus therapy. During clinical trials, patients who began sirolimus with normal, fasting, total serum triglycerides developed hypertriglyceridemia after treatment with sirolimus 2 mg and 5 mg. All patients treated with sirolimus should be monitored for hyperlipidemia. If detected, treatment with diet, exercise, and lipid-lowering drug should be initiated as described by the National Cholesterol Education Program guidelines (Prod Info Rapamune(R), 2001; MacDonald et al, 2001).Dose-dependent HYPERTRIGLYCERIDEMIA and to lesser extent HYPERCHOLESTEROLEMIA occurred with the use of sirolimus as primary immunosuppression or after its addition to cyclosporine-based regimens in renal transplant patients (MacDonald et al, 2001; Groth et al, 1998; Slaton et al, 1995; Kahan, 1998; Brattstrom et al, 1998; Murgia et al, 1996). The hyperlipidemia associated with cyclosporine/steroids is enhanced by addition of sirolimus, increasing the risk of cardiovascular events and possibly graft rejection. In one study (n=26), triglyceride levels exceeding 5 mmol/L were seen in 75% of patients receiving various doses of sirolimus in addition to cyclosporine-based regimens, with mean peak levels of 14.5 mmol/L occurring after a mean of 7 weeks. Parallel increases in total cholesterol were observed, but were less pronounced (mean peak, 12 mmol/L). In patients receiving trough level-controlled sirolimus, a reduction in target trough levels from 30 to 15 ng/mL after two months led to a decrease in triglyceride levels. Triglyceride levels returned to pretreat mentstatus in all patients receiving sirolimus after 6 months (Brattstrom et al, 1998).

Dose reduction of sirolimus is indicated during the first few months posttransplantation if excessive rises in triglyceride (e.g., 10 mmol/L) and cholesterol are observed. Discontinuation should be considered in the face of high triglyceride levels (eg, 20 mmol/L),

particularly in diabetics. The use of pravastatin or fibrates during first month has been advocated (Brattstrom et al, 1998). A postulated mechanism of hyperlipidemia is inhibition of insulin and insulin-like growth factor signals or alterations in hepatocyte synthesis or distribution of lipid moieties (Kahan, 1998).

METABOLIC EFFECTS

During clinical trials, HYPOPHOSPHATEMIA occurred in 15% to 23% of patients receiving sirolimus, while HYPOKALEMIA occurred in 11% to 21% of patients (Prod Info Rapamune(R), 2001). Diabetogenic effects of sirolimus were observed in early clinical studies (Hughes & Gruber, 1996). Hyperglycemia has not been reported as a complication in subsequent studies.

GASTROINTESTINAL

GASTROINTESTINAL EFFECTS

During clinical trials, gastrointestinal adverse events including CONSTIPATION (28% to 36%), DIARRHEA (25% to 42%), DYSPEPSIA (17% to 25%), NAUSEA (25% to 36%), and VOMITING (19% to 25%) were reported (Prod Info Rapamune(R), 2001).

KIDNEY/GENITOURINARY

RENAL EFFECTS

During phase III clinical trials that used combination therapy with sirolimus and cyclosporine, the mean serum creatinine was increased while the mean glomerular filtration rate was decreased. These effects were NOT observed with cyclosporine and placebo or azathioprine controls. Therefore, renal function should be carefully monitored in patients treated with this combination. If the serum creatinine rises, appropriate adjustments should be made to the immunosuppression regimen (Prod Info Rapamune(R), 2001).

GENITOURINARY EFFECTS

URINARY TRACT INFECTION occurred in 20% to 33% of patients receiving sirolimus (Prod Info Rapamune(R), 2001).

LIVER

HEPATIC EFFECTS

Overall, oral sirolimus has not significantly affected liver function (as assessed by transaminase changes) when added to cyclosporine-based regimens in renal transplant patients (Murgia et al, 1996; Slaton et al,1995). However, isolated elevations in both alanine and aspartate transaminase occurred with low or high doses. Results of larger, long-term studies are needed to adequately assess hepatotoxic potential.

RESPIRATORY

RESPIRATORY EFFECTS

Progressive INTERSTITIAL PNEUMONITIS was reported in 3 renal transplant recipients receiving sirolimus. Patients were also receiving concomitant immunosuppressants and lipid-lowering agents. Dose and duration of therapy were not addressed. All 3 patients had chest films and computed tomography showing bilateral pulmonary infiltrates. Two of the 3 patients were symptomatic with exertional dyspnea, and arterial blood gas measurements revealing hypoxemia.

No evidence of infection was found with bronchoalveolar lavage, but a lymphocytic pneumonitis was noted in 2 of the patients. Sirolimus therapy was withdrawn (other medications remained unchanged), and the patients' clinical and radiologic status improved within a few weeks. No antibiotics were initiated, and the interstitial pneumonitis resolved within 3 months in all patients (Morelon et al, 2000).

DYSPNEA has been reported in 22% to 30% of patients receiving sirolimus during clinical trials (Prod Info Rapamune(R), 2001). In a phase III trial, 9% of patients receiving sirolimus 5 milligrams (mg)/day (n=208) and 3% of patients receiving sirolimus 2 mg/day (n=219) experienced EPISTAXIS versus none of the patients receiving placebo (n=124). All patients received cyclosporine and corticosteroids (MacDonald et al, 2001).

MUSCULOSKELETAL

MUSCULOSKELETAL EFFECTS

ARTHRALGIA (25% to 31%) occurred at a higher incidence in patients treated with sirolimus versus azathioprine (Prod Info Rapamune(R), 2001). In a phase III trial, 23% of patients receiving sirolimus 5 milligrams (mg)/day (n=208) and 16% of patients receiving sirolimus 2 mg/day (n=219) reported arthralgia's versus 11% receiving placebo (n=124). All patients received cyclosporine and corticosteroids (MacDonald et al, 2001).

OTHER

ADVERSE EFFECTS - GENERAL

INFECTION

In a phase III trial, 19% of patients receiving sirolimus 5 milligrams (mg)/day, 10% of patients receiving sirolimus 2 mg/day, and 8% of patients receiving placebo reported mucosal HERPES SIMPLEX infections. All patients received cyclosporine and corticosteroids (MacDonald et al, 2001). The overall incidence of bacterial, viral, or fungal infections due to EXCESSIVE IMMUNOSUPPRESSION has not increased significantly with addition of sirolimus to cyclosporine regimens in recipients of cadaveric or living kidneys (Kahan, 1998). In particular, no effect on the incidence/severity of cytomegalovirus disease has been observed.

POSTTRANSPLANT LYMPHOPROLIFERATIVE DISEASE

Posttransplant lymphoproliferative disease (PTLD) has been uncommon with addition to sirolimus to cyclosporine, occurring in 2 of 250 evaluable renal transplant patients (0.8%) treated for 3 months to 2 years in one series (Kahan, 1998). In both cases, this effect of excessive immunosuppression was reversed by discontinuation of sirolimus and cyclosporine dose reduction.

TERATOGENICITY/EFFECTS IN PREGNANCY

TERATOGENICITY

U.S. Food and Drug Administration Pregnancy Category C (Prod Info Rapamune(R), 2001).

**3.1.2	Pharmaceutical Data (For details of Pharmacokentics please see Package Insert from the PDR)**

3.1.3 **Administration**

The appropriate dose of sirolimus oral solution should be measured using an amber oral syringe. The dose should be added to a glass or plastic container that holds at least 60 milliliters (mL). Before taking the dose, it should be diluted with water or orange juice; IT SHOULD NOT BE DILUTED WITH GRAPEFRUIT JUICE. After mixing with the diluent, the dose should be taken immediately. About 120 mL of the diluent should be added to the container and mixed vigorously. This fluid should also be taken immediately (Prod Info Rapamune(R), 2001).

Early clinical studies used formulations containing dimethylacetamide (DMA), whereas this component has been omitted in more recent studies; the pharmacokinetics of sirolimus are similar with each vehicle (Yatscoff et al, 1995a). A new oral formulation with improved bioavailability and an intravenous formulation are under development.

Oral doses should be administered consistently with regard to meals (e.g., with or without) (Prod Info Rapamune(R), 2001).

**3.1.4	Storage and Stability**

Sirolimus oral solution should be protected from light and should be stored in the refrigerator between 2 and 8 degrees Celsius (C) (36 to 46 degrees Fahrenheit (F)). Once opened the solution should be used within 1 month. If necessary, the opened container may be stored at room temperature up to 25 degrees C (77degrees F) for up to 30 days (Prod Info Rapamune(R),

2001).

The tablets should be stored at 20 to 25 degrees Celsius (C) (68 to 77 degrees Fahrenheit (F)). The tablets should be protected from light (Prod Info Rapamune(R), 2001).

**4.0	ELIGIBILITY CRITERIA**

4.1 Patients must have an intracranial Glioblastoma multiforme, as confirmed on pathological examination of the biopsy by Dr. Paul S. Mischel., a board certified Neuropathologist.
4.2 Patient’s whose tumors demonstrate at least 25% focal loss of PTEN staining by immunohistochemical analysis will be eligible.
- PTEN immunohistochemistry will be performed on paraffin-embedded biopsies using the anti-PTEN monoclonal antibody 6H2.1 (Cascade Biosciences, Winchester, MA) using conditions optimized in the laboratory of Dr. Paul S. Mischel.
- Appropriate positive and negative controls will be performed. In addition, GFAP and MIB-1 antibodies will be used. These markers will provide additional diagnostic information and verify that the paraffin-embedded biopsy tissue is suitable for immunohistochemical analysis.
- All PTEN immunohistochemical staining will be analyzed by Dr. Paul S. Mischel using the following system:
  a) PTEN staining intensity will be graded into three groups: ++ equals staining of tumor cells that is equal to, or greater than, staining in vascular endothelial cells (which serves as an internal control); + equals diminished staining of tumor cells relative to vascular endothelial cells; - equals no tumor cell staining.
  b) In addition, because PTEN loss can be heterogeneous, tumors that demonstrate loss of PTEN staining (+ or – intensity) in greater than, or equal to 25% of tumor cells in the biopsy will be eligible.
  4.3 All patients must have shown unequivocal evidence for tumor progression by MRI or CT.
      4.3.1 Patients with prior therapy that included interstitial brachytherapy or stereotactic radiosurgery must have confirmation of true progressive disease rather than radiation necrosis based upon either PET, Thallium scanning, MR spectroscopy or surgical documentation of disease.

**4.4**	Registration Imaging studies

**4.4.1.**Modified Phase I patients must be eligible for a salvage surgery for enrollment. There are no set requirements for the registration imaging study.

**4.4.2.**Phase II portions of the study, patients with recurrent disease must have shown unequivocal evidence for tumor progression by MRI or CT scan. This scan should be performed within 14 days prior to registration. The same type of scan, i.e., MRI or CT must be used throughout the period of protocol treatment for tumor measurement.

**4.5	Phase II** Patients having undergone recent resection of recurrent or progressive tumor will be eligible as long as all of the following conditions apply:

**4.5.1.**	They have recovered from the effects of surgery.

**4.5.1.1.**	To best assess the extent of residual disease post-operatively, a CT/ MRI should be done no later than 96 hours in the immediate post-operative period or, if not obtained with in 96 hours post operatively, 4-6 weeks post-operatively.

**4.5.1.2.**	The baseline on-study MRI/CT is performed within 14 days prior to registration.

4.6 For both the phase Ib and II portions, patients with progressive disease must have failed prior radiation therapy and must have an interval of greater than or equal to 4 weeks from the completion of radiation therapy to study entry.
4.7. Prior Therapy guidelines:
     4.7.1. **For the modified phase I component patients are eligible any time after the first relapse.**
     4.7.2. For the phase II component, patients with recurrent malignant glioma may have had treatment for no more than 3 prior relapses.

**Relapse represents a time at which the tumor has progressed after receiving some definitive therapy such as surgery, radiation, chemotherapy, biologic agent , and/or experimental therapy.**

4.8. All patients must sign an informed consent indicating that they are aware of the investigational nature of this study.
4.9. Patients must be ≥ 18 years old.
4.10. Patients must have a life expectancy > 8 weeks.
4.11. Patients must have a Karnofsky performance status of ≥ 60.
4.12. Patients must have recovered from the toxic effects of prior therapy **and** at least two weeks from vincristine, 6 weeks from nitrosoureas, 3 weeks from procarbazine administration, and 1 week for non-cytotoxic agents, e.g., interferon, tamoxifen, etc. (radiosensitizer does not count).
4.13. Patients must have adequate bone marrow function (WBC ≥ 3,000/µl, ANC ≥ 2,000/mm^3^, platelet count of ≥ 100,000/mm^3^, and hemoglobin ≥ 10 gm%) and test must be performed within 14 days prior to registration. Eligibility level for hemoglobin may be reached by transfusion.
4.14. Patients must have adequate liver function (SGOT and bilirubin < 1.5 times ULN), **and cholesterol level must be <350 mg/dl and triglycerides < 400mg/dl.** These tests must be performed within 14 days prior to registration.
4.15. Patients must have adequate renal function (creatinine < 1.5 mg/dL and creatinine clearance ≥ 60 mL/min- measured or estimated) before starting therapy and tests must be performed within 14 days prior to registration.
4.16. Patients must not have any significant medical illnesses that in the investigator’s opinion cannot be adequately controlled with appropriate therapy or would compromise the patients’ ability to tolerate this therapy.
4.17. Patients with a history of any other cancer (except non-melanoma skin cancer or carcinoma in-situ of the cervix), unless in complete remission and off of all therapy for that disease for a minimum of 3 years are ineligible.
4.18. This study was designed to include women and minorities, but was not designed to measure differences of intervention effects. Males and females will be recruited with no preference to gender. No exclusion to this study will be based on race. Minorities will actively be recruited to participate.
4.19. Patients must not have active infection.
4.20. Patients must not become pregnant and must practice adequate contraception. The effects rapamycin on the developing human fetus are unknown. For this reason, women of child-bearing potential and men must agree to use adequate contraception (hormonal or barrier method of birth control) prior to study entry, for the duration of study participation. Should a woman become pregnant or suspect she is pregnant while participating in this study, she should inform her treating physician immediately.
4.21. Patients must not have disease that will obscure toxicity or dangerously alter drug metabolism.
4.22. Patients must not have serious inter-current medical illness.
4.23. Patients must not have history of allergic reactions attributed to compounds of similar chemical or biologic composition to rapamycin,
4.24. Patients must give the drug name(s) prior to registration, if he or she received prior therapy.

5.0 **STRATIFICATION/DESCRIPTIVE FACTORS**
    5.1 Since all patients will not be on EIAEDs we will not stratify base upon use of anticonvulsant use.

**6.0	TREATMENT PLAN**

**6.1	General**

All patients who meet eligibility criteria must be registered with UCLA - Neuro-oncology Program clinical trials office.

6.2 **Starting Dose:** The starting dose for patients in phase Ib and anticipated in Phase II will be 6mg loading dose on day one and 2mg/day. .
6.3 **Treatment Schedule**

**Modified Phase I study:**

Initial dose of rapamycin will be 6mg on day one and then 2 mg each day for 5-7 days before surgery. The patient will then come off drug while recovering from surgery. When the patient has fully recovered from surgery the patient will then initiate rapamycin with a 6 mg loading dose on day one and then 2 mg each day. For the purpose of evaluation, a cycle will be defined as every 4 weeks. Repeated cycles will be administered assuming that there are no dose-limiting toxicities or clinical deterioration. During the 8^th^ week of treatment patients undergo clinical and radiographic tumor restaging. As long as the tumor is stable or smaller in size and the patient is clinically stable or improved, they will go on to another 2 four week cycle of therapy with Rapamycin. Treatment will continue indefinitely as long as there are no unacceptable toxicities (DLTs) and no tumor progression.

Patients in the Phase II will receive the same dosing scheme unless the tissue results from Phase Ib do not reach the anticipated goals.

The following dose levels will be used:

| **Dose Level** | **Dose (mg)** |
| --- | --- |
| **3**  **2**  **1 (starting dose)**  **-1**  **-2** | **21 mg load/7 mg/day**  **15 mg load/5 mg/day**  **6 mg load/2 mg /day**  **4 mg load/1.5 mg/day**  **3 mg load/1 mg/day** |

Although the goal of this study is to define the dose at which S6 kinase is blocked in 3/3 patient tumors, we will also evaluate the patient for toxicity in each cohort and if MTD is reached before a dose that defines biologic efficacy then the phase II study will be held and further investigation into other drug schedules will take place.

Once 3/3 patient show blocking of S6 Kinase, three additional patients will be enrolled. Tumor samples in these patients will also need to show inhibition of S6 Kinase. Drug levels will be obtained as described. The results of these drug levels will provide guidance with regard to dose adjustments allowed in the phase II study.

**MTD:**

The MTD will be based on the tolerability observed during the first 4 weeks of treatment only. Escalations are planned in groups of three patients, with an additional three patients to be added at the first indication of Dose Limiting Toxicity (DLT). The MTD of Rapamycin will be that dose at which less than one-third of patients experience DLT. If dose level 3 is reached and the MTD has not been established, subsequent escalation may continue at 33% increase of the prior dose.

Up to three patients may be enrolled simultaneously at each dose level. These patients should be observed for DLT for at least 4 weeks from the first day of treatment before new patients are enrolled at the next higher dose level. **If patients are not fully evaluable for toxicity in cycle 1, patients will be replaced.**

The following dose escalation rules will be used:

1) Three patients are studied at the first dose level.
2) If none of these three patients experience DLT, then the dose is escalated to the next higher level in the three subsequent patients.
3) If one of the three patients experience DLT at the current dose, then three more patients are accrued at the same dose. If none of these three additional patients suffer DLT, then the dose is escalated in subsequent patients. If one or more of these three additional patients experiences DLT, the MTD has been exceeded and three more patients are treated at the next lower dose (if only three patients were previously treated at that prior dose).
4) If two or more patients encounter DLT, then the MTD has been exceeded and three more patients are treated at the next lower dose level (if only three patients were previously treated at that dose level).
5) The MTD is the dose at which 0/3 or 1/6 patients experience DLT with the next higher dose having at least 2/3 or 2/6 patients encountering DLT.
6) **Dose limiting toxicity (DLT):** Toxicities will be graded according to the NCI Common Toxicity Criteria (CTC Version 2.0) scale. If multiple toxicities are seen, the presence of DLT should be based on the most severe toxicity experienced. DLT will be defined as any of the following events occurring during the first 4 week course of treatment of CCI-779 and attributable to the study drug:
   a. **Grade 4 neutropenia that lasts more than 5 days, febrile neutropenia and any other grade 4 hematologic toxicity**
   b. **Grade 4 hypercholesterolemia, hyper triglyceridemia**
   c. Any other non-hematologic grade 3 toxicity.
   d. Failure to recover from toxicities to be eligible for re-treatment with rapamycin within 2 weeks of the last dose of rapamycin treatment.

**Phase II study:**

Once the biologically effective dose has been established, this dose will be used for the phase II portion of the study in this patient cohort. Patients will be treated at the defined phase II dose first with a loading dose and then daily in repeated cycles where a cycle is defined as 28 days or 4 weekly doses. Repeated cycles will be administered assuming that there are no dose-limiting toxicity or clinical deterioration. Alterations in the dose will be allowed based upon Drug levels obtained during the modified Phase I study. These ranges of dose alterations will be determined in consultation with the statistician. During the 8^th^ week of treatment patients undergo clinical and radiographic tumor restaging. As long as the tumor is stable or smaller in size and the patient is clinically stable or improved, they will go on to another 2 four week cycle of therapy with rapamycin. Treatment will continue indefinitely as long as there are no unacceptable toxicities (DLTs) and no tumor progression.

6.4 Surgical Treatment Plan: Patients who are candidates for surgical resection of recurrent disease and are eligible for the phase Ib component of the study will be considered for a pre-operative study to evaluate biological/tissue correlates. Patients will be administered the agent as a loading dose and then daily treatment for 5-7 days prior to scheduled surgery. In addition to the acquisition of recurrent tumor at the time of surgical resection, whole blood samples (to be placed in 7 ml EDTA tubes **and in 7 ml Red top tubes for serum**) will be drawn at the time of tumor resection . See section 8.6 for tissue processing and storage. Whole blood should be stored as outlined in Section 8.5.

Post-operative evaluations as a result of this part of the study will consist of routine postoperative care.

6.6 Once patients have recovered from the effects of surgery, they will embark upon the daily dosing of the study drug. Patients are not required to have measurable or evaluable disease to continue onto the daily dosing phase.
6.7 Patients will be monitored for hematologic or serologic evidence of myelosuppression, hepatic injury, renal injury, and electrolyte disturbances and for clinical evidence of other toxicity as is described in the study calendar.
6.8 Growth Factors: Routine prophylactic use of G-CSF is not permitted. However, therapeutic use in patients with serious neutropenic complications such as sepsis, may be considered at the investigator’s discretion.
6.9 Supportive Care:
    6.9.1 Corticosteroids should be used in the smallest dose to control symptoms of cerebral edema and mass effect, and discontinued if possible.
    6.9.2 Anti-seizure medications should be used as indicated. Every effort should be made to keep the patients off of EIAED, however, if a patient is changed to a EIAED for greater than ten days the patient will be withdrawn from the study.
    6.9.3 Febrile neutropenia may be managed according to the local institution's Infectious Disease guidelines. Measures may include appropriate laboratory testing, including blood and urine cultures and the institution of broad-spectrum antibiotics. If a source for the fever is not identified or the fever resolves when the neutrophil count recovers, antibiotics may be discontinued and the patient observed.
    6.9.4 If neurosurgical management is required for reasons not due to tumor progression, these procedures must be documented, including the indications for surgery, the surgical operative note and pathology report.
6.10 **Dose Modifications**
     6.10.1 Patients with stable or responding disease may be retreated at the same dose or at a reduced dose level, depending upon the adverse events observed in the current cycle and any adverse events present on the first day of the next cycle. If multiple toxicities are seen, the dose administered in a subsequent cycle should be based on the most severe toxicity experienced in the current cycle. Dose modifications or delays should be made based upon whether toxicities occur within a 4-week treatment cycle or at the expected start of the next treatment cycle.
     6.10.2 Within treatment cycles: If a patient experiences DLT (as defined in Section 6.3) during the current treatment cycle, the treatment will be immediately suspended for a minimum of 1 week. If the toxicity resolves to less than or equal to grade 1 within 2 weeks, the patient will be retreated at one dose level lower. Patients experiencing DLT at the initial starting dose there will be a dose reduction to the next lower level. **One** further dose reduction is allowed **to the next dose level.** Doses that are reduced for rapamycin related toxicity will not be re-escalated, even if there is minimal or no toxicity with the reduced dose. Patients whose dose has been reduced for adverse events that are subsequently not felt to be related to rapamycin may have the dose re-escalated after completion of one cycle with toxicities less than or equal to grade 1.
     6.10.3 At the start of the next treatment cycle:

**Dose Modifications:** The National Cancer Institute Common Toxicity Criteria Scale, version 2 (copies will be provided to the sites), will be used to grade toxicity. If a patient experiences a toxicity determined to be at least possibly related to rapamycin administration, the dose administered for subsequent weeks may require a reduction of the dose, depending on the grade and type of toxicity observed.

**Dose Modifications Based on Weekly ANC and Platelet Counts:**

**ANC (/mL)	Platelets (/mL)	% of Planned CCI-779 Dose**

> 1000	and	> **80,000**	100%

750-999	or 50,000 to < **80,000**	hold*

< 750	or	< 50,000	hold**

*Upon recovery to ANC ³ 1,000/mL and platelets to ³ 100,000/mL, 75% of dose will be administered.

** Upon recovery to ANC ³ 1,000/mL and platelets to ³ 100,000/mL, 50% of dose will be administered.

**Dose Modifications Based on every month cholesterol and triglycerides**

**NCI Grade	% of Planned Rapamycin Dose**

0-3 100%

4	hold	.

* use of cholesterol lowering agent may be used to treat AE related to cholesterol or triglycerides

**Dose Modifications Based on Rapamycin-Related Non- Hematologic Toxicities:**

**NCI Grade	% of Planned Rapamycin Dose**

0-2+	100%+

3*	hold**

4	hold***

+	For symptomatic Grade 2 toxicity, the dose may be held until recovery to CTC Grade 0-1, then 75% of the dose administered, at the investigator's discretion.

*	Except nausea/vomiting (unless patients are on optimal antiemetic therapy)

**	Hold until recovery to CTC Grade 0-2 (or to within 1 grade of starting values for pre-existing laboratory abnormalities), and then 75% of dose will be administered.

***	Hold until recovery to CTC Grade 0-2 (or to within 1 grade of starting values for pre-existing laboratory abnormalities), and then 50% of dose will be administered.

Patients will be allowed two dose reductions. If any patient has further toxicities that would require further reduction, or if treatment is held for more than 2 consecutive weeks because of ongoing toxicity, **the investigator will assess if the patient should remain in the study if the patient has benefited from treatment.**

A new course of treatment may begin when the ANC is at least 1000/mm3 and the platelet count is at least 100,000/mm3 and any other treatment-related toxicities are less than or equal to grade 1. If after a one-week delay, all toxicities are less than or equal to grade 1, then proceed with treatment at the dose level dictated by the modifications outlined above. If toxicities have not resolved to less than or equal to grade 1 after a one-week delay, then treatment will be held again, and the patient will be evaluated weekly. If treatment will be held for 2 weeks, then reduce the rapamycin dose by one dose level beyond that indicated by whether or not the patient had DLT in the prior cycle. If re-treatment must be held for more than 2 weeks **the investigator should be notified.** If a patient develops another DLT at the reduced dose or if they experience a life-threatening toxicity at any time, they will be removed from study.

6.11 For treatment or dose modification related questions, please contact the principle investigator.

**7.0	PRETREATMENT EVALUATION**

7.1 A complete history and neurological examination, as well as documentation of evaluable disease shall be performed on all patients. The treating physician will review the scan done prior to study entry. Baseline scan should be performed within 14 days of registration.

Pre-study laboratory tests shall include CBC, platelets, PT, PTT, serum creatinine, creatinine clearance (measured or estimated), bilirubin, SGOT, albumin, alkaline phosphatase, total bilirubin, bicarbonate, BUN, calcium, chloride, glucose, phosphorus, potassium, sodium, serum **cholesterol and** triglycerides and serum pregnancy test for women of childbearing potential. Anticonvulsant levels that are monitored will also be measured. Pre-study laboratory tests must be obtained with 14 days of registration.

8.0 **EVALUATION DURING STUDY**
    8.1 **Evaluations unique to modified Phase I. Patients will be able to restart study drug once:**
        8.1.1 They have recovered from the effects of surgery.
        8.1.2 To best assess the extent of residual disease post-operatively, a CT/ MRI should be done no later than 96 hours in the immediate post-operative period or, if not obtained with in 96 hours post operatively, 4-6 weeks post-operatively.
        8.1.3 The baseline on-study MRI/CT is performed within 14 days prior to restarting study drug.
        8.1.4 **N**eurological examination, as well as documentation of evaluable disease shall be performed on all patients.
        8.1.5 Prior to restarting study drug laboratory tests shall include CBC, platelets, PT, PTT, serum creatinine, creatinine clearance (measured or estimated), bilirubin, SGOT, albumin, alkaline phosphatase, total bilirubin, bicarbonate, BUN, calcium, chloride, glucose, phosphorus, potassium, sodium, serum **cholesterol and** triglycerides and serum pregnancy test for women of childbearing potential. Anticonvulsant levels that are monitored will also be measured. Pre-study laboratory tests must be obtained with 14 days of restarting drug.
    8.1 CBC and platelets and Sirulimus levels will be performed every week during treatment for patients on the Phase Ib portion of the study. For patients on the phase II portion, weekly CBC and platelets and sirulimus leves will be obtained for the first cycle and every 2 weeks thereafter.

**Serum level for tumor markers will be performed at baseline prior to start the drug, at the time of the surgery, then at two weeks on the treatment, at eight weeks on the treatment then every 8 weeks.** Creatinine, or creatinine clearance, bilirubin, SGOT, calcium, glucose, **cholesterol and** triglycerides will be performed every two weeks prior to each cycle. Anticonvulsant levels will be measured on day 1 of cycle 1 and cycle 2 and then every other cycle.

8.2 Vitals, PS, neuro exam and brain MRI/CT will be done prior to every other cycle. Perfusion MRI, magnetic resonance spectroscopy (MRS) will be requested but is not mandatory (as some third party payers may refuse to reimburse for these studies). Patients achieving a response of PR or CR will be evaluated by the PI.
8.3 Every attempt will be made to obtain a baseline PET scan. A repeat PET scan will be performed at 2 weeks and at 8 weeks.
8.4 All relevant information regarding drug doses, concomitant medications, and doses, measurable lesions with measurements, tumor response, laboratory examinations, and treatment-related toxicities shall be documented in the patient’s medical record and flow sheets.
8.5 All patients will be followed for overall survival, when possible.
8.6 Pharmacokinetic evaluation. The major pharmacokinetic goal of this study is to determine the pharmacokinetic characteristics of rapamycin in glioma patients.

Every effort should be made to collect all samples at times specified. Samples missed or lost for any reason should be recorded. Both the scheduled and actual times of blood collection should appear on the blood collection record used at the clinic. The time of the start and finish of the infusion should also be recorded.

Phase Ib: blood sample will be obtained at time of surgery for rapamycin levels

**Cycle 1- All patients:**

**Blood sample Times (all will be trough levels):**

Day 8	Prior to daily dose of rapamycin

Day 15

Day 22

Cycle 2, Day 1	Blood samples will occur every two weeks for cycle 2-4 and then will occur every cycle

The total volume of blood collected for the pharmacokinetic assessments (6 specimens x 7ml) is 42 ml during the first two months of the study.

**Specimen Collection Procedures and Handling Instructions:**

**Whole blood (for drug assay) and for serum tumor markers:**

**Blood samples will be evaluated through the UCLA Clinical laboratories.**

**Collect blood samples (7ml) in Lavender top tube for the drug assay and collect blood samples 7 ml in Red top tube for serum tumor markers.**

**Patient labels will be applied to the tubes.**

**Samples in the Red top tube should be sent to Dr. Charles Sawyers lab.**

**8.7	Surgical Specimens:**

In the Phase Ib portion of the study, some patients may undergo craniotomy or stereotactic biopsy for recurrent tumor. Tumor specimens not required for histologic diagnosis will be divided into 2 fragments when feasible. Two fragments will be frozen **immediately** in liquid nitrogen and stored at –70 degrees C.

One fragment frozen in liquid nitrogen will be used to measure the concentration of rapamycin in the tumor.

### Dr. Charles Sawyers

Another fragment frozen in liquid nitrogen will be used to measure the following by western blot technique:

PTEN

PAkt

Total S6K

Phosphorylated S6K

Total 4E-BP1(PHAS-1)

Phosphorylated 4E-BP1 (PHAS-1)

**All patients in the phase Ib and Phase II study will be required to have Unstained paraffin Slides or tissue blocks evaluated prior to enrollment:** Unstained Paraffin slides will be obtained in all phase Ib/II study patients from original surgery or most recent surgery and in those patients who will be undergoing resection for recurrent tumor. Paraffin slides will be used to perform the following studies:

PTEN-IHC

EGFR

**MIB-1**

These samples should be sent to:

### Paul Mischel, MD

675 Charles Young Drive South

MacDonald Research Laboratories, Room 4579

Los Angeles, CA 90095

**9.0	CRITERIA FOR EVALUATION AND ENDPOINT DEFINITIONS**

9.1 **Endpoints for the modified Phase I Portion of the study**
    9.1.1 **The primary endpoint will be to determine the dose that rapamycin blocks S6 kinase phosphorylation.**
    9.1.2 **A secondary endpoint will be the description of the toxicities of rapamycin in patients on and determination of MTD if the primary endpoint is not met.**
    9.1.3 **Another secondary endpoint will be the determination of the pharmacokinetics of rapamycin. This will allow for the determination of range on dose adjustments allowed in the phase II portion of the study**
9.2 **Endpoints for the Phase II portion of the study**
    9.2.1 For patients with recurrent malignant glioma, the primary endpoint will be progression free survival (PFS) at six months.
    9.2.2 Secondary endpoints include:

a. Toxicity

b. Tumor response as assessed by neuro-imaging and neurologic exam

c. Survival

d. Pharmacokinetics

9.3 **RECIST criteria:** Criteria permitting uni-dimensional response measurement have recently been adopted and published by an international consensus committee, Response Evaluation Criteria In Solid Tumors Group (RECIST). In order to ensure comparability of data from this trial with data from earlier trials, this trial will retain the traditional evaluation definition in Section 9.3 as primary. However, RECIST criteria that can be collected, will be used for secondary evaluation. Concordance with the traditional approach will be summarized.

**9.3	Definitions of Response**

9.3.1 **Measurable Disease:** Bi-dimensionally measurable lesions with clearly defined margins by CT or MRI scan.
9.3.2 **Evaluable Disease:** Uni-dimensionally measurable lesions, masses with margins not clearly defined. Patients with only this kind of imaging will not be allowed to enter this study.
9.3.3 **Non-Evaluable Disease:** Not Applicable for response evaluation.
9.3.4 **Objective Status, To Be Recorded at Each Evaluation:** If there are too many measurable lesions to measure at each evaluation, choose the largest two to be followed before a patient is entered on study. The remaining lesions will be considered evaluable for the purpose of objective status determination. Unless progression is observed, objective status can only be determined when ALL measurable and evaluable sites and lesions are assessed.
      9.3.4.1 **Complete Response (CR):** Complete disappearance of all measurable and evaluable disease. No new lesions. No evidence of non-evaluable disease. All measurable, evaluable and non-evaluable lesions and sites must be assessed using the same techniques as baseline. Patients must be on minimal i.e. adrenal replacement doses or no steroids.
      9.3.4.2 Part**ial Response (PR):** Greater than or equal to 50% decrease under baseline in the sum of products of perpendicular diameters of all measurable lesions. No progression of evaluable disease. No new lesions. All measurable and evaluable lesions and sites must be assessed using the same techniques as baseline. Responders must be on the same or decreasing doses of dexamethasone.
      9.3.4.3 **Partial Response, Non-Measurable (PRNM):** Not applicable.
      9.3.4.4 **Stable/No Response (NR)/No Change (NC):** Does not qualify for CR, PR, or progression. The designation of Stable/No Response requires a minimum of 8 weeks duration. All measurable and evaluable sites must be assessed using the same techniques as baseline.
      9.3.4.5 **Progression:** 25% increase in the sum of products of all measurable lesions over smallest sum observed (over baseline if no decrease) using the same techniques as baseline, OR clear worsening of any evaluable disease, OR appearance of any new lesion/site, OR failure to return for evaluation due to death or deteriorating condition (unless clearly unrelated to this cancer).
      9.3.4.6 **Unknown:** Progression has not been documented and one or more measurable or evaluable sites have not been assessed.

**9.4	Best Response:** This will be calculated from the sequence of objective statuses

For patients with all disease sites assessed every evaluation period, the best response will be defined as the best objective status as measured according to Section 10.2. If the response does not persist at the next regular scheduled MRI, the response will still be recorded based on the prior scan, but will be designated as a non-sustained response. If the response is sustained, e. g. still present on the subsequent MRI, it will be recorded as a sustained response, lasting until the time of tumor progression. Best response is unknown if the patient does not qualify for a best response or increasing disease and if all objective status determinations before progression are unknown.

**9.5	Neurological Exam:** Although not used for determining response, it is useful to evaluate improvement in the neurologic exam that should coincide with objective measurement of tumor size.

9.5.1 Normal exam.
9.5.2 Improvement from on-study state (patient better).
9.5.3 Same as on-study state (patient same).
9.5.4 Worse than on-study state (patient worse).

**9.6	Performance Status, Neurological Function, and Mental Status:** Patients will be graded according to Karnofsky Performance Status**,** Neurological Function and Mental Status (see Appendix 18.2).

**9.7	Time to Treatment Failure:** From date of registration to the date of first observation of progressive disease (as defined in Section 9.3.4.5), non-reversible neurologic progression or permanently increased steroid requirement (applies to stable disease only), death due to any cause, or early discontinuation of treatment.

**9.8	Time to Death:** From date of registration to date of death due to any cause.

**10.0	CRITERIA FOR REMOVAL FROM STUDY**

**10.1	Criteria for Removal from Protocol Treatment:**

10.1.1 Progression of disease (as defined in Section 9.3.4.5).

Patients must be followed by the same type of brain scan, as was used for baseline tumor measurements, and will be removed from study if progression is documented after any cycle of treatment. Patients with stable disease, partial or complete response will continue on therapy as defined in Section 6.3.

10.1.2 Unacceptable toxicity (see Section 6.3 ).
10.1.3 The patient may withdraw from the study at any time for any reason.
10.1.4 Medical or psychiatric illness which in the investigator's judgment renders the patient incapable of further therapy.

**10.2	All reasons for discontinuation of treatment must be documented in the flow sheets.**

**11.0	STATISTICAL CONSIDERATIONS**

**11.1	Phase I study analysis:**

Baseline characteristics will be summarized across all enrolled patients. An accounting will be done of all patients registered in the study. The number of patients who died or withdrew before treatment began will be specified. Patients who did not meet all eligibility criteria will be described. Patients who were evaluated separately due to first-cycle non-drug-related death or cessation of first-cycle therapy prior to DLT will be characterized. **Patients not fully evaluable for toxicity in cycle 1 will be replaced.**

Treatment administration will be described for all cycles. Doses administered, dose modifications or delays, and duration of therapy will be evaluated.

Safety variables will be summarized by descriptive statistics. Adverse events that occur will be reported for each dose level and described in terms of incidence and severity. Laboratory data will be presented by dose level at each observation time. Values outside of normal limits will be identified and their frequency calculated. Parameters will be described based on the CTC severity grading. Distribution by CTC severity grade (when applicable) and clinical relevance will be given.

A descriptive analysis of evidence of antitumor activity will be provided based on clinical, radiographic, and biologic assessments of efficacy.

Bioanalytical and Pharmacokinetic Analysis: The whole blood concentration of rapamycin will be quantified by LCMS/MS method.

Whole blood concentration of rapamycin will be evaluated and analysis will determine concentration ranges that will be used for allowable dose adjustments in phase II study.

The anticipated rate of modified phase I accrual will be 3 patients/3months, until the biologic endpoint or MTD is established. If a total of 12 patients are enrolled, the enrollment period will be 12 months. **It is estimated that there will be 6-18 patients enrolled.**

**11.2	Phase II Design and Analysis Plan**

**11.2.1**	Recurrent Malignant Glioma subset

For patients with recurrent malignant glioma, the primary endpoint of this phase of the study will be to determine if rapamycin can significantly delay progression in patients with recurrent malignant glioma. The primary endpoint is progression-free survival at 6 months. The trial cohort will include GBM only. The trial will be sized to be able to discriminate between a 15% and 35% rate for the GBM group alone. With accrual of 32 GBM patients, the trial will be considered a success if at least 8 GBM patients show 6-month progression-free survival. This will give a 0.92 probability of detecting a 35% rate of 6-month progression-free survival, with 0.9 probability of rejecting the agent if the 6-month progression-free survival is only 15%.

The secondary endpoint in this cohort is the response rate associated with rapamycin therapy in recurrent or progressive malignant glioma. This will be accomplished by determining the proportions of patients experiencing complete or partial response and stable disease as defined in Section 9.3.4. With 32 GBM patients, the maximal width on a 95% confidence interval would be 0.35. The exact 95% confidence intervals of 0.5 would be (0.32, 0.68) for 32 patients. The exact 95% confidence intervals for 0.05 would be (0.01, 0.21) for 32 patients (2/32=6%).

**11.3	Statistical Analysis of Biologic Correlate Data**

**11.3.1	Tumor Tissue Assays:** We will estimate the mean or median difference in assay values between the primary resection and the recurrence resection for patients with tissue from both resections (these will be paired data). We will also estimate the mean or median differences between the assay values in treated and untreated patients (these will be unpaired data). If sufficient data are available, we will adjust these differences for relevant covariates. Parametric or nonparametric methods will be used depending on the observed distribution of the data (in terms of skewness or outliers).

**12.0	DISCIPLINE REVIEW**

**12.1	Pathology Review**

**12.1.1**	Following registration, slides from the pre-registration biopsy must be submitted for review. The purpose of this review is to verify the histologic diagnosis and to determine PTEN status

The materials are to be submitted prior to registration to:

**Paul Mischel, MD**

**12.1.2**	Pathology Materials Required for Review

12.1.2.1 One to two representative H&E stained slides from a pre-registration biopsy demonstrating lesion(s).
12.1.2.2 A copy of each the Pathology Report and Operative Report.
12.1.2.3 Protocol Specific Pathology Submission Form.
12.2 **Radiology Review**
     12.2.1 The central review of neuro-imaging (MRI or CT) will be performed at UCLA on registered patients who have been determined by the enrolling institution as responders. Responders will include CR or PR.
     12.2.2 All films of all views from pre-registration and subsequent scans must be submitted for central review by the UCLA

**13.0	ANALYTICAL ASSAY**

Plasma concentrations of rapamycin will be determined by a validated LC/MS/MS method.

14.0 **LABORATORY CORRELATES**

See section 8.0 for detailed descriptions.

15.0 **Data Safety Monitoring Board**
     15.1 **This protocol will use the JCCC Data Safety Monitoring Board. We will abide by the rules and procedures set forth by this DSMB.**
     15.2 **Safety Assessments and Toxicity Monitoring**

**All patients receiving Rapamycin will be evaluated for safety. The safety parameters include all laboratory tests and hematological abnormalities, CNS observations, physical examination findings, and spontaneous reports of adverse events reported to the investigator by patients. All toxicities encountered during the study will be evaluated according to the NCI Common Toxicity Criteria version 2.0 in Section 17.1 and recorded prior to each course of therapy. Life-threatening toxicities should be reported immediately to the study Coordinator (Principal Investigator), Institutional Review Board (IRB), ISPRC , JCCC DSMB and the Food and Drug Administration (FDA).**

**A serious adverse event is any adverse drug experience at any dose that results in any of the following outcomes: Death, a life-threatening adverse drug experience, inpatient hospitalization or prolongation of existing hospitalization, a persistent or significant disability/incapacity, or a congenital anomaly/birth defect. Important medical events that may not result in death, be life-threatening, or require hospitalization may be considered serious when, based upon appropriate medical judgment, they may jeopardize the patient or may require medical or surgical intervention to prevent one of the outcomes listed in this definition. Examples of such medical events include allergic bronchospasm requiring intensive treatment in an emergency room or at home, blood dyscrasias or convulsions in a patient who has never had seizure activity in the past that do not result in inpatient hospitalization, or the development of drug dependency or abuse.**

**All Serious Adverse Events ,regardless of attribution will be reported to the IRB and JCCC DSMB within 5 days of discovery using the Adverse Event report Form for UCLA and Non UCLA Reports.**

**The study will utilize CTEP Common Toxicity Criteria (CTC) Version 2.0 for Toxicity and Adverse Event reporting. A copy of the CTC version 2.0 can be downloaded from the CTEP home page (****http://ctep.info.nih.gov)****. All appropriate treatment areas should have access to either a hard copy of the CTC version 2.0 or the www version.**

**15.3	Quality Assurance**

**Quality assurance measures are provided by three mechanisms: ongoing monitoring of protocol compliance, on-site audits, and response reviews. Monitoring will begin at the time of patient registration and will continue during protocol performance and completion. This trial is subject to Cancer Center Internal Quality Assurance Monitoring.**

**Data and the progress of this trial will be reported to the JCCC DSMB at regular intervals as determined by the JCCC DSMB. The first data summary report will be due quarterly, starting from the date of the IRB approval.**

**16.0	REFERENCES**

1) Ali IU, Schriml LM, Dean M. Mutational spectra of PTEN/MMAC1 gene: a tumor suppressor with lipid phosphatase activity. JNCI 1999; 91, 1922-1932.
2) Akoki M, Blazek E, and Vogt PK. A role of the kinase mTOR in cellular transformation induced by the oncoproteins P3k and Akt. PNAS 2000;98(1): 136-141.
3) Brunn GJ, Hudson CC et al. Phosphorylation of the translational repressor PHAS-1 by the mammalian target of rapamycin. Science 1997, 277 :99-101.
4) Cantley LC, Neel BG. New insights into tumor suppression: PTEN suppresses tumor formation by restraining the phosphoinositide 3-kinase/AKT pathway. PNAS 1999;96: 4240-4245.
5) Gingras A, Kennedy SG et al. 4E-BP1, a repressor of mRNA translation, is phosphorylated and inactivated by the Akt(PKB) signaling pathway. Genes Dev 1998 12:502-13.
6) Hashemolhosseini S, Nagamine Y et al. Rapamycin inhibition of the G1 to S transition is mediated by effects on cyclin D1 mRNA and protein stability. J Biol Chem 1998. 273:14424-14429
7) Maehama T, Dixon JE. PTEN: a tumor suppressor that functions as a phospholipid phosphatase. Trends Cell Biol 1999; 9: 125-128.
8) Metcalfe SM, Canman CE et al. Rapamycin and p53 act on different pathways to induce G1 arrest in mammalian cells. Oncogene 1997. 15: 1635-42.
9) Molnar-Kimber KL. Mechanism of action of rapamycin. Transplant Proc 1996. 28 :964-9
10) Muise-Helmericks RC, Grimes HL et al. Cyclin D expression is controlled post-transcritionally via a phosphatidylinositol 3-kinase/Akt-dependent pathway. J Biol Chem 1998. 45: 29864-72.
11) Neshat MS, Mellinghoff IK, Tran C, Stiles B, Thomas G, Wu H, Sawyers CL. Enhanced sensitivity of PTEN-deficient tumors to inhibition of mTOR. (submitted).
12) Seghal SN, Molnar-Kimber KL, Ocain TD et al. Rapamycin; a novel immunosuppressive agent. Med Res Rev. 1994 14:1.
13) Seufferlein T, Rozengurt E. Rapamycin inhibits constitutive p70s6k phosphorylation, cell proliferation and colony formation in small cell lung cancer cells. Cancer Res 1996. 56: 3895-7.
14) Takuwa N, Fukui Y, and Takuwa Y. Cyclin D1 expression mediated by phosphatidylinositol 3-kinase through mTOR-p70(S6K)-independent signaling in growth factor-stimulated NIH 3T3 fibroblast. Mol Cell Biol 1999 Feb; 19(2): 1346-58.
15) Wang SI, Puc J, Li J et al. Somatic mutations of PTEN in glioblastoma multiforme. Cancer Res 1997. 57:4183.
16) Wiederrecht GJ, Sabers CJ et al. Mechanism of action of rapamycin:new insights into regulation of G1-phase progression in eukaryotic cells. Prog Cell Cycle Res 1995. 1:53-71.
17) Geoerger B, Kerr K, Tang C-B et al. Antitumor activity of the Rapamycin analog CCI-779 in human primitive neuroectodermal tumor/medulloblastoma models as single agent and in combination chemotherapy. Can Res 2001, 61:1527-1532.

**17.0	APPENDIX**

**17.1	NCI Common Toxicity Criteria**

This study will utilize the CTC version 2.0 for Toxicity and Adverse Event reporting. A copy of the CTC version 2.0 can be downloaded from the CTEP home page (http://ctep.info.nih.gov).

**17.2	Karnofsky Performance Status, Neurological Function, and Mental Status**

Patient's performance status will be graded according to the following scale:

**17.2.1	Karnofsky Performance Status**

| **KPS 100** | Normal; no complaints; no evidence of disease |
| --- | --- |
| **KPS 90** | Able to carry on normal activity; minor signs or symptoms of disease |
| **KPS 80** | Normal activity with effort; some sign or symptoms of disease |
| **KPS 70** | Cares for self; unable to carry on normal activity or do active work |
| **KPS 60** | Requires occasional assistance, but is able to care for most personal needs |
| **KPS 50** | Requires considerable assistance and frequent medical care |
| **KPS 40** | Disabled; requires special care and assistance |
| **KPS 30** | Severely disabled; hospitalization is indicated, although death no imminent |
| **KPS 20** | Very sick; hospitalization necessary; active support treatment is necessary |
| **KPS 10** | Moribund; fatal processes progressing rapidly |
| **KPS 0** | Dead |

**17.2.2	Neurological Function**

| **0** | No neurologic symptoms; fully active at home/work without assistance |
| --- | --- |
| **1** | Minor neurologic symptoms; fully active at home/work without assistance |
| **2** | Moderate neurologic symptoms; fully active at home/work but requires assistance |
| **3** | Moderate neurologic symptoms; less than fully active at home/work and requires assistance |
| **4** | Severe neurologic symptoms; totally inactive requiring complete assistance at home or institution; unable to work |

**17.2.3	Mental Status**

| **0** | Normal function |
| --- | --- |
| **1** | Minor mental confusion |
| **2** | Gross confusion but awake |
| **9** | Unknown |

**17.3.0** **Model Informed Consent**

17.3.1 **Study Calendar**

| **Data Set** | **Study**  **Entry** | **Q wk** | **Q 2wks** | **2^nd^**  **Cycle** | **Q 8 wks** |
| --- | --- | --- | --- | --- | --- |
| **Physical Exam** | **X** |  |  |  | **X** |
| **Performance Status** | **X** |  |  |  | **X** |
| **Neurological Exam** | **X** |  |  |  | **X** |
| **History** | **X** |  |  |  |  |
| **CBC** | **X** | **X ^a, b^** | **X ^c^** |  |  |
| **Platelet Count** | **X** | **X ^a, b^** | **X ^c^** |  |  |
| **PT** | **X** |  |  |  |  |
| **PTT** | **X** |  |  |  |  |
| **Serum Creatinine** | **X** |  | **X ^c^** |  |  |
| **Bilirubin** | **X** |  | **X ^c^** |  |  |
| **Total Bilirubin** | **X** |  | **X ^c^** |  |  |
| **SGOT** | **X** |  | **X ^c^** |  |  |
| **Albumin** | **X** |  | **X ^c^** |  |  |
| **Glucose** | **X** |  | **X ^c^** |  |  |
| **Alkaline Phasphatase** | **X** |  | **X ^c^** |  |  |
| **Bicarbonate** | **X** |  | **X ^c^** |  |  |
| **BUN** | **X** |  | **X ^c^** |  |  |
| **Na^+^** | **X** |  | **X ^c^** |  |  |
| **Cl^-^** | **X** |  | **X ^c^** |  |  |
| **K^+^** | **X** |  | **X ^c^** |  |  |
| **Ca^2+^** | **X** |  | **X ^c^** |  |  |
| **Phosphorus** | **X** |  | **X ^c^** |  |  |
| **Triglyceride/cholesterol** | **X** |  | **X ^c^** |  |  |
| **Pregnancy Test** | **X** |  |  |  |  |
| **Pharmacokinetic (PK)** | **X** | **X ^e^** | **X ^e^** | **X** |  |
| **Serum tumor marker** | **X** h |  | **X** |  | **X** |
| **MRI or CT Scan** | **X** |  |  |  | **X** |
| **MRS (Optional)** | **X** |  |  |  | **X** |
| **Surgical specimen** | **X ^f^** |  |  |  |  |
| **Pathology determination of Molecular inclusion** | **X** |  |  |  |  |
| **Anti-Convulsant Level** | **X ^d^** |  |  | **X ^d^** | **X ^d^** |
| **PET Scan** | **X** |  | **X ^g^** |  | **X** |

a:	CBC and platelet will be performed every week during the treatment for patients on the Phase I portion of the study.

B:	CBC and platelet will be performed weekly for the first cycle and every 2 weeks thereafter during the treatment for patients on the Phase II portion of the study.

C:	Tests will be performed every 2 weeks prior to each cycle.

D:	Anti-convulsant levels will be measured on day 1 of cycle 1 and cycle 2 and then every other cycle.

E:	During the first cycle rapamycin blood levels will be obtained each week. After the first cycle the blood levels will be obtained every two weeks.

F:	Some patients may undergo craniotomy or stereotactic biopsy in the Phase II portion of the study. Tumor specimen will be obtained and handled by following the instructions provided in Section 8.6.

g:	2 week PET scan will only occur in the second week of the first cycle.

H : serum tumor markers will be performed prior the first dose prior the surgery, at the surgery, prior restarting the treatment, at 2 weeks, at 8 weeks on the treatment then every 8 week.

**17.4.0 The List of EIAEDs and Non-EIAEDs**

| **Non-EIAEDs** |  | **EIAEDs** |
| --- | --- | --- |
| Valproic acid |  | Carbamazapine |
| Gabapentin |  | Oxcarbazepine* |
| Lamotrigine |  | Phenytoin |
| Topriamate |  | Fosphenytoin |
| Levetiracetam |  | Phenobarbital |
| Tiagabine |  | Primidone |
| Zonisamide |  |  |
| Clonazepam |  |  |

* Enzyme induction limited and less potent than Carbamazapine.
